# DRDB: A Machine Learning Platform to Predict Chemical-Protein Interactions towards Diabetic Retinopathy

**DOI:** 10.1155/2022/1718353

**Published:** 2022-07-20

**Authors:** Yu Wei, Ruili Zhang, Xiaoqiang Li, Zhonglin Li, Kaimin Guo, Shanshan Li, Li Yan, Qian Zhao, Baijian Qu, Wenjia Wang, Shuiping Zhou, He Sun, Jianping Lin, Yunhui Hu

**Affiliations:** ^1^State Key Laboratory of Medicinal Chemical Biology, Frontiers Science Center for Cell Responses, College of Pharmacy and Tianjin Key Laboratory of Molecular Drug Research, Nankai University, Tianjin 300353, China; ^2^Cloudphar Pharmaceuticals Co., Ltd., Shenzhen, China; ^3^The State Key Laboratory of Core Technology in Innovative Chinese Medicine, Tasly Academy, Tasly Holding Group Co., Ltd., No. 1, Tingjiang West Road, Beichen District, Tianjin 300410, China

## Abstract

Diabetic retinopathy (DR), a diabetic microangiopathy caused by diabetes, affects approximately 93 million people, worldwide. However, the drugs used to treat DR have limited efficacy and the variety of side effects. This is possibly because the complicated pathogenesis of DR is associated with multiple proteins. In this work, we attempted to identify potential drugs against DR-associated proteins and predict potential targets for drugs using in silico prediction of chemical-protein interactions (CPI) based on multitarget quantitative structure-activity relationship (mt-QSAR) method. Therefore, we developed 128 binary classifiers to predict the CPI for 15 DR targets using random forest (RF), *k*-nearest neighbours (KNN), support vector machine (SVM), and neural network (NN) algorithms with MACCS, extended connectivity fingerprints (ECFP6) fingerprints, and protein descriptors. In order to facilitate discovery of the novel drugs and target identification using the 128 binary classifiers, a free web server (DRDB) was developed. Compound Danshen Dripping Pills (CDDP), composed of Salvia miltiorrhiza, Panax notoginseng, and borneol, is commonly used in the treatment of cardiovascular diseases. To explore the applicability of DRDB, the potential CPIs of CDDP in treatment of DR were investigated based on DRDB. In vitro experimental validation demonstrated that cryptotanshinone and protocatechuic acid, two key components of CDDP, are capable of targeting ICAM-1 which is one of the key target of DR. We hope that this work can facilitate development of more effective clinical strategies for the treatment of DR.

## 1. Introduction

Diabetic retinopathy (DR) is one of the most important manifestation of diabetic microangiopathy, which is a fundus disease with specific changes and one of the serious complications of diabetes [1]. Worldwide, there are approximately 93 million DR patients. Diabetes patients mainly suffer from abnormal insulin hormones and cell metabolism, which cause changes in eye tissue, nerve and blood vessel microcirculation, and damage eye nutrition and visual function. Due to the change of blood composition in diabetic patients, the function of vascular endothelial cells is abnormal, and the blood-retinal barrier is damaged [2, 3]. The lesions of retinal capillaries include aneurysms, bleeding spots, hard exudates, cotton wool spots, beaded veins, intraretinal microvascular abnormalities (IRMA), and macular edema.

Current treatments for DR include drug therapy, laser photocoagulation, and vitrectomy [4]. In terms of drug treatment, there are drugs to control blood sugar, lower blood lipids, and control blood pressure [5]. In earlier studies, doxium (calcium 2, 5-dihydroxybenzene sulfonate) was found to significantly inhibit and reverse the three high factors leading to DR: high capillary permeability, high blood viscosity, and high platelet activity [6]. Aspirin also has a certain preventive effect on microthrombosis, which can inhibit the production of thromboxane and prostaglandin metabolites and inhibit the platelet agglutination [7]. Therapeutic drugs were divided into four categories, including antivascular endothelial growth factor (anti-VEGF), corticosteroids, angiotensin receptor blockers, and fibrates drugs. However, the current therapies for DR are associated with the limited efficacy and the variety of side effects. Topical nonsteroidal anti-inflammatory drugs have proven to be ineffective in long-term diabetic macular edema (DME) treatment [8]. Besides, intravitreal antivascular endothelial growth factor drugs may increase the risk of cardiovascular complications in diabetic patients [8]. Therefore, there is a need to develop the effective treatment or more efficacious drugs.

Disease progression of DR is associated with multitargets as a complicated disease. Currently, target prediction and identification of optimal candidates in drug discovery strongly depend on computational intelligence and data-driven decision. As for target prediction and identification of optimal candidate, identification of the chemical-protein interaction (CPI) between proteins and chemicals is crucial. Compared to traditional experimental identification, in silico computational approaches are time-saving and low-cost. Several types of drug-related interactions have received an enormous amount of attention recently. Chen et al. summarized the databases, web servers, and state-of-the-art computational models (e.g., network-based method and machine learning-based method) involved in CPI [9]. The advantage of most of state-of-the-art computational models is that they are suitable for compounds without known 3D target structures, and they are applicable to predict novel CPI for new compounds. For pathway-based drug discovery, the new strategy of identification of the drug-pathway associations is developed based on various state-of-the-art computational methods including matrix decomposition-based, Bayesian spare factor-based, and some machine learning methods [10]. In addition, microRNAs have been identified as diagnostics and therapeutic targets in recent years [11]. The state-of-the-art computational methods have been developed to identify the potential small molecule-miRNA associations. Recently, Koutsoukas et al. summarized the computational methods for predicting the CPI, including ligand-based approach and structure-based approach [12]. Multitarget quantitative structure-activity relationship (mt-QSAR) method, as a ligand-based approach, facilitates the prediction of activities against different proteins and exploration of multiple pharmacological activities. To explore the polypharmacology against DR, construction of multiclassifiers for target prediction is appreciated [13].

**Figure 1:**
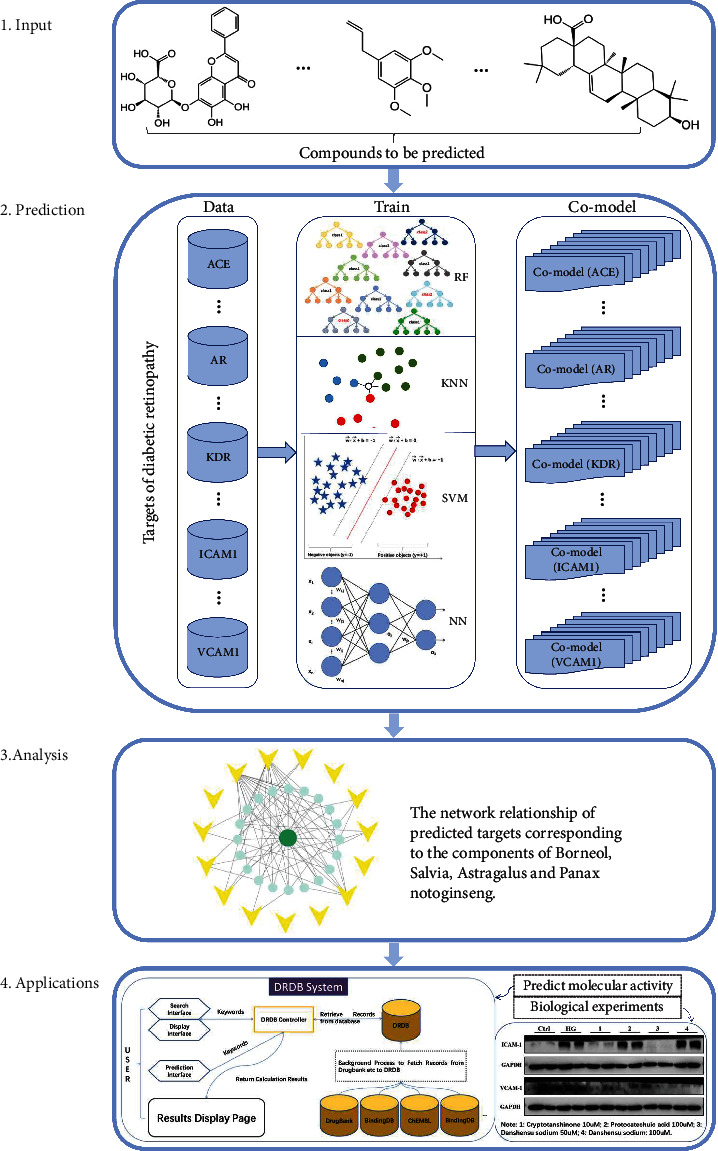
The workflow of DRDB server to predict CPIs towards DR.

To apply the mt-QSAR method to predict CPI towards DR, 15 targets (ACE, AGTR1, FLT1, PRKCB, AKR1B1, AR, ICAM1, MAPT, NOS2, NOS3, SERPINE1, SLC2A1, TNF, VCAM1, and KDR) involved in the progression of DR were selected to construct the binary classifiers in this work. We constructed multiple classifiers based on random forest (RF), *k*-nearest neighbours (KNN), support vector machine (SVM), and neural network (NN) algorithms with MACCS, ECFP6 fingerprints, and protein descriptors. After that, multiple classifiers of each target were integrated into a platform for systematic target prediction in a comprehensively DR database (http://tangwang.tasly.com/). To evaluate the applicability of DR database, we collected the components of Salvia miltiorrhiza, Panax notoginseng, and borneol from CDDP and applied the binary classifiers manufactured in DR database to predict the potential targets for these components. Then, system pharmacology-based investigation of Salvia miltiorrhiza, Panax notoginseng, and borneol against DR were conducted. The prediction results were further confirmed by in vitro experimental validation. The schematic workflow of DRDB implementation is presented in Figure 1.

## 2. Materials and Methods

### 2.1. Data Collection and Processing for Classification Model Construction

DR-associated genes were collected and integrated from the databases including DisGeNET, OMIM, and Genecard. High-frequency genes were analyzed by literature retrieval, in which 15 important genes (including ACE, AGRT1, FLT1, PRKCB, AKR1B1, AR, ICAM1, MAPT, NOS2, NOS3, SERPINE1, SLC2A1, TNF, VCAM1, and KDR) were finally screened out to build QSAR modelling.

The biological activity of 15 genes was collected from ChEMBL database [14]. The compounds with specific IC_50_ values were selected to construct the binary classifiers for each target. And the compounds were preprocessed as follows: (i) duplicated compounds in each set were removed; (ii) salts were neutralized; (iii) compounds were classified into active and inactive categories according to pIC_50_ (-log IC_50_ (mol/L)) values based on the criteria that obtained a balanced distribution of active and inactive compounds. The details of each data set used to construct the predictive model are presented in Table S1. The preparation of data set is carried out using the software KNIME 4.1.0 (https://www.knime.org).

### 2.2. The Construction of Random Forest, *K*-Nearest Neighbours, Support Vector Machine, and Neural Network Models

The small molecules were characterized using MACCS fingerprint and extended connectivity fingerprints (ECFP), respectively. The MACCS and ECFP6 (1024 dimensional counted with a radius set to 3) fingerprints were computed using RDKit. For each target, data set was divided into a training set (80% data set) and a test set (20% data set) using the stratified sampling method. Training set was used to train the models, and 5-fold cross-validation was performed for internal model validation. Test set was used as an external dataset to evaluate the quality of the model. Eight predictive models (RF_MACCS, RF_ECFP6, SVM_MACCS, SVM_ECFP6, KNN_MACCS, KNN_ECFP6, NN_MACCS, and NN_ECFP6) were constructed for each target based on MACC and ECFP6 fingerprints and four machine learning algorithms (random forest (RF) [15], *k*-nearest neighbours (KNN) [16], support vector machine (SVM) [17], and neural network (NN) [18]).

To investigate the influence of the factors of proteins in modelling performance, proteochemometrics (PCM) was implemented by integrated descriptors of chemical compounds with descriptors of proteins. The PyBioMed [19] was used to calculate the amino acid composition, transition, and distribution descriptors for 15 targets. In total, 8568 protein descriptors were calculated for each target sequence. The values of all of descriptors were normalized in the range between 0 and 1 using the min-max normalization function. The principal component analysis (PCA) was used to reduce the dimensions of protein descriptors. After that, 150 protein descriptors were preserved and used to construct the PCM models. Training and test sets for PCM modelling were constructed by combining data sets of 15 targets. Then, 12289 chemicals (positives: 6137, negatives: 6152) and 3080 chemicals (positives: 1539, negatives: 1541) constitute the integrated training and test sets for the PCM models, respectively. Eight predictive models (RF_MACCS_protein, RF_ECFP6_protein, SVM_MACCS_protein, SVM_ECFP6_protein, KNN_MACCS_protein, KNN_ECFP6_protein, NN_MACCS_protein, and NN_ECFP6_protein) were constructed based on the composition of chemical compounds and descriptors of proteins.

Random forest (RF), an ensemble method, is consisted of many decision trees which produce individual predictions. The most votes of a large number of trees determine the classification result of RF, which has become a “gold standard” with high prediction accuracy for the comparison with other machine learning methods [20].

*K*-nearest neighbours (KNN) make predictions using proximity by grouping the individual data point. The value *k* refers to the number of closest neighbours that are used in the voting process. For classification problems, a majority voting rule is used to assign a class label by counting the class of *k* closest neighbours.

Support vector machine (SVM) is developed based on Vapnik's structural risk minimization (SRM) principle of the statistical learning theory and is applicable for dealing with nonlinear problems for classification by constructing a hyperplane to separate positive and negative samples with a maximum margin.

Neural network (NN) comprises several neurons which are connected to each other and organized into layers. NN attempts to identify the potential relationships in input data through mimics the study of human brain and utilize complicated mathematical models for processing information.

To evaluate the prediction capability of the predictive models, five indicators including sensitivity (SE), specificity (SP), accuracy (Q), Matthews correlation coefficient (MCC), and area under curve (AUC) were used. These indicators were calculated based on the true positives (TP), true negatives (TN), false positives (TP), and false negatives (FN) in the following way:

(1) $$\begin{array}{c} \text{SE}=\frac{\text{TP}}{\text{TP}+\text{FN}}, \end{array}$$

(2) $$\begin{array}{c} \text{SP}=\frac{\text{TN}}{\text{TN}+\text{FP}}, \end{array}$$

(3) $$\begin{array}{c} Q=\frac{\text{TP}+\text{TN}}{\text{TP}+\text{TN}+\text{FP}+\text{FN}}, \end{array}$$

(4) $$\begin{array}{c} \text{MCC}=\frac{\text{TP}\times \text{TN}-\text{FN}\times \text{FP}}{\sqrt{\left(\text{TP}+\text{FN}\right)\left(\text{TP}+\text{FP}\right)\left(\text{TN}+\text{FN}\right)\left(\text{TN}+\text{FP}\right)}}. \end{array}$$

### 2.3. Database Implementation

DR-related chemicals and drugs were extracted from ChEMBL and DrugBank databases, respectively. The signaling pathways associated with 15 targets and DR-related drugs were extracted from KEGG database. HERB (http://herb.ac.cn) was used to collect natural compounds. We manually checked records from DrugBank, ChEMBL, KEGG, Pharmacodia, and TTD, to obtain up-to-date information about DR drugs (update to February 1, 2022). The approved or clinical trial drugs for DR treatment were stored in DRDB database. Finally, by searching ChEMBL ID of 15 DR-related protein targets, a list of chemicals, reported bioactivities, bioassays, references, and drugs for the treatment of other diseases associated with proteins were retrieved and stored in the backend of DRDB.

The DR database's client and RESTful server sides were built with the Angular web framework and the Django REST framework (http://www.django-rest-framework.org), respectively. DRDB database was installed using PostgreSQL (http://postgresql.org) on an Ubuntu server. The RDKit package (http://rdkit.org), an open source cheminformatics toolkit, was used for similarity search and prediction. Furthermore, the JSME Javascript plugin was used to draw structures on the website.

### 2.4. Meta-Analysis

China National Knowledge Infrastructure (CNKI), WanFang, VIP, and PUBMED databases were electronically searched to collect all relevant publications that reported Compound Danshen Dripping Pills treating DR by using the following search terms: “Compound Danshen Dripping Pills” and “Diabetic retinopathy.” The literature search was performed up to November 2021. All statistical analyses were performed using the Review Manager Software (RevMan5.4) provided by Cochrane. The weighted mean differences (WMD) of the measurement data are used as the combined statistic, of which 95% confidence intervals (CI) were assessed, and the forest map was made [21]. Heterogeneity was assessed by the WHAT test, *I*^2^ > 50% or *P* < 0.1 is used to assess significance, and a random effects model is used to explain the possible causes of heterogeneity. If *I*^2^ < 50%, there is no heterogeneity, and a fixed effects model will be used [22]. The magnitude of publication bias is judged by the degree of symmetry of the funnel graph [23]. The information of inclusion criteria, exclusion criteria [24–27], data extraction [28–42], and study quality assessment [43] could be referred in supporting information.

### 2.5. Collection of Components of Salvia miltiorrhiza, Panax notoginseng, and Borneol

YaTCM [44] is a free web-based Chinese medicine database, which contains 6,220 herbal medicines, 47,696 natural compounds, and 18,697 targets. Different from TCMSP [45], ETCM [46], HERB [47], and SymMap [48], herbal medicines are included in YaTCM. YaTCM can be obtained free of charge at http://cadd.pharmacy.nankai.edu.cn/yatcm/home. SymMap, a comprehensive Chinese medicine database enhanced by symptom mapping, contains 499 kinds of medicinal materials and 19,595 kinds of ingredients registered in the Chinese Pharmacopoeia. The monomer components of three herbs (Salvia miltiorrhiza, Panax notoginseng, and borneol) in CDDP were extracted from the YaTCM, HERB, and SymMap databases and represented as SMILES format according to their PubChem CID. In total, there are 69 ingredients in borneol, 261 ingredients in Salvia miltiorrhiza, and 354 ingredients in Panax notoginseng.

### 2.6. Target Prediction for Components of Salvia miltiorrhiza, Panax notoginseng, and Borneol and Chemical-Protein Interaction Network Analysis

We applied multiple classifiers of each target to predict the putative targets for molecules against DR. In order to take the advantage of different classifiers, the multiple voting method was applied to estimate whether a compound was active against a target. The more classifiers predict to be positive, the more likely it is considered as a valid chemical-protein interaction. To explore the possible mechanism of borneol, Salvia miltiorrhiza, and Panax notoginseng, we constructed the compound-target network. The potential chemical-protein interaction network was constructed and analyzed by Cytoscape 3.9.1 (Cytoscape Consortium, United States) software.

### 2.7. Target Validation In Vitro

The targets predicted by QSAR model were validated in vitro. Cells were cultured in Dulbecco's Modified Eagle's medium/F12 medium (DMEM/F12; Gibco) supplemented with 10% foetal bovine serum (Life iLab Biotech) and 1% penicillin/streptomycin at 37°C in a humidified incubator with 5% CO_2_. In order to establish a hyperglucose cell model, ARPE-19 cells were treated with 50 mM D-glucose for 72 hours. In addition, ARPE-19 cells were treated with cryptotanshinone and protocatechuic acid (10 mM, 20 mM) for 72 hours in the presence of high glucose. Cells cultured in DMEM without glucose served as the control. Cell viability were measured using Cell Counting Kit-8 (CCK-8) after drug treatment.

When the cells reached the logarithmic growth phase, the medium was replaced with the serum-free medium containing different drugs, and the cells were continued to be cultured for 72 hours. To extract the total protein, cells were collected and disrupted with RIPA lysis buffer (Solarbio) and ultrasonic processor. The supernatant was obtained after centrifugation, which concentration was estimated with Bicinchoninic Acid Protein Assay kit (Thermo Fisher Scientific, Inc.). 40 *μ*g protein from each sample was separated on 10% sodium dodecyl sulfate polyacrylamide gels. The samples were transferred onto nitrocellulose filter membrane (Millipore), which were then blocked with 5% skim milk for one hour at room temperature. After blocking, the membrane was incubated overnight at 4°C with appropriate primary antibodies (GAPDH, 1 : 8000, Proteintech; ICAM-1, 1 : 500, Santa Cruz Biotechnology). The next day, membrane was washed three times with TBST buffer and then incubated with HRP-conjugated secondary antibodies (1 : 5000; KPL) at room temperature for two hours. The membrane was washed three times again to wash off the residue antibody solution completely and interacted with enhanced chemiluminescence substrate (Millipore). Protein band was detected with chemiluminescence gel imaging system (Tanon 5200).

## 3. Results and Discussion

### 3.1. Machine Learning Models

The classification performance of 120 classifiers for 15 targets was evaluated using various metrics, and the results are presented in Tables 1 and 2. In the cross-validation process, the MCC values of 86 classifiers out of 120 (71.67%) are greater than 0.5, 86 models out of 120 (71.67%) give an AUC value higher than 0.75. In general, AUC values of 120 models are greater than 0.667 with an average value of 0.782, and *Q* valuesare greater than 0.676 with an average value of 0.783, indicating that the models have reasonable classification performance (Table 1). In additions, test set was used to further evaluate the performance of the classifiers, and the results are listed in Table 2. As presented in Table 2, the MCC values range from 0.192 to 0.944, with an average value of 0.590. The AUC values range from 0.596 to 0.971, with an average of 0.791. Among the 15 targets, eight classifiers from the microtubule-associated protein tau (MAPT) did the worst performance, with the average AUC and MCC values of 0.69 and 0.39, respectively. Perhaps the main reason for this is due to few compounds (*n* = 71) included in the training set, which limits to a narrow application domain of classifiers target MAPT. For most of targets, the prediction results obtained with ECFP6 are better than that obtained with MACCS.

The PCM classifiers for each combination of fingerprints and protein descriptors (MACCS and protein descriptors, ECFP6 and protein descriptors) were constructed using RF, SVM, KNN, and NN as well. In total, eight classifiers for each target were developed. The performance of each classifiers were evaluated by 5-fold cross-validation and test set. Statistical characteristics of these models can be found Tables 1 and 2, respectively. PCM classifiers achieve an average AUC of 0.783 and 0.794 on training and test data separately, which is comparable to results from eight classifiers obtained with molecular fingerprints (MACCS and ECFP6). Similar to eight classifiers obtained with molecular fingerprints (MACCS and ECFP6), the performance of PCM classifiers based on the combination of ECFP6 and protein descriptors is better than that of PCM classifiers based on the combination of MACCS and protein descriptors.

In this investigation, 16 classifiers (RF_MACCS, RF_ECFP6, SVM_MACCS, SVM_ECFP6, KNN_MACCS, KNN_ECFP6, NN_MACCS, NN_ECFP6, RF_MACCS_protein, RF_ECFP6_protein, SVM_MACCS_protein, SVM_ECFP6_protein, KNN_MACCS_protein, KNN_ECFP6_protein, NN_MACCS_protein, and NN_ECFP6_protein) of each target were used to select compounds with potential inhibitory activity against the corresponding target. Because different combinations of fingerprints and machine learning algorithms have different prediction performance, we used the multivoting ensemble method to predict CPIs. Then, we further evaluated the prediction performance of the multivoting ensemble method based on votes of 16 classifiers, and cutoff was defined as the number of voting classifiers (ranging from 1 to 16) giving positive label. The statistical results of the multivoting ensemble method on integrated test set (3080 samples) are presented in Table 3. Results from Table 3 present that with the increase of cutoff, SP is increasingly from 0.491 to 0.962, and SE is decreasingly from 0.957 to 0.409. The results indicate that the lower cutoff, a larger acquisition ability of positives and a lower differentiated ability of negatives. However, the higher cutoff is more likely to identify negatives with the greater loss of positives. The best prediction results of multivoting ensemble method were achieved with cutoff = 9, resulting in AUC = 0.824, *Q* = 0.824, and MCC = 0.648.

In addition, we compared the prediction performances of multiple machine learning classifiers with DeepConv-DTI, which predicts the drug-target interactions via deep learning with convolution based on protein sequences [49]. For keeping the consistency of the training and test sets, the same training and test sets for PCM models were used to train and evaluate DeepConv-DTI model. Hyperparameters are listed as follows: (i) learning rate is 0.0001; (ii) the number of epochs is 50; (iii) the batch size is 32; (iv) activation function is exponential linear unit (ELU) [50]. For DeepConv-DTI model, the AUC, SE, SP, and *Q* of integrated test set are 0.878, 0.812, 0.796, and 0.804, respectively. We observe that DeepConv-DTI model is comparable to PCM models based on RF, SVM, and KNN algorithms in terms of AUC, SE, SP, and Q, and it is significantly better than PCM model-based NN algorithm. Compared to multivoting ensemble method, DeepConv-DTI model does not provide significant predictive advantages in this study.

### 3.2. DRDB Interface

**Figure 2:**
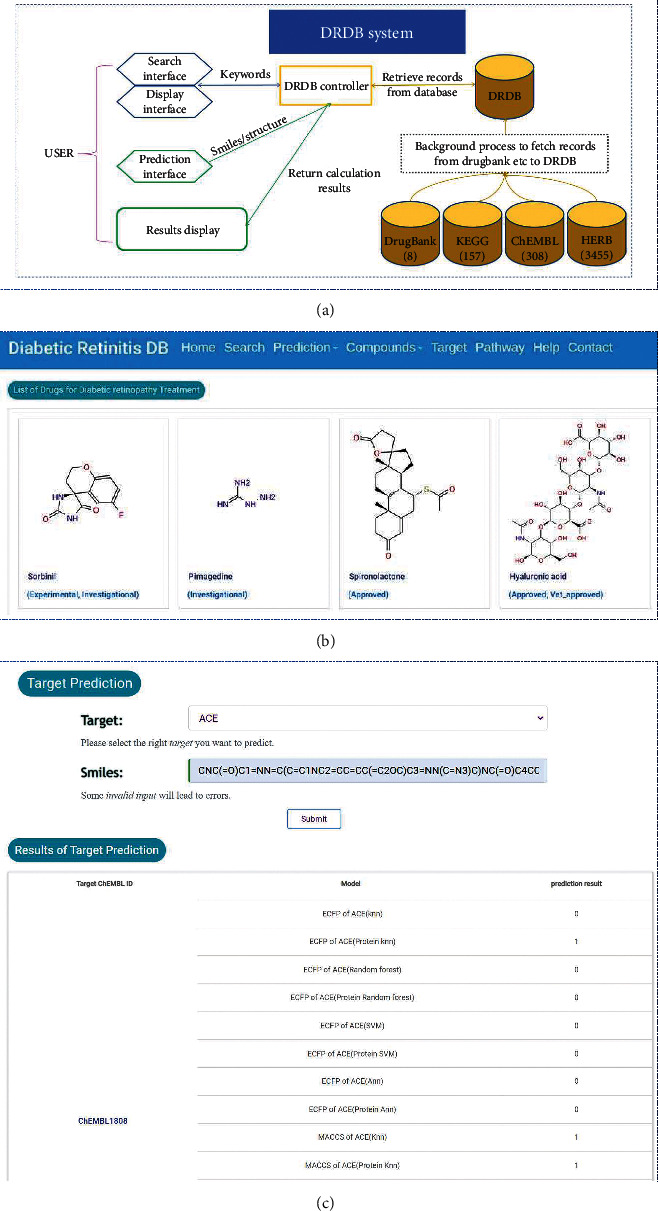
Overview of DRDB database featured with integrated computing and data-mining functions. (a) 15 genes, 157 pathways, 8 approved drugs, 308 chemicals, and 3455 ingredients are included in the DRDB. (b) Display interface of DRDB database. (c) Overview of the application of the DRDB database for target prediction.

In this investigation, multiple binary classifiers based on mt-QSAR method for 15 targets are constructed and integrated into a DR chemogenomics database—DRDB. DRDB is available for free on the internet at http://tangwang.tasly.com/. It is advised to use the most recent versions of browsers, such as Firefox or Chrome. In DRDB, 15 genes, 157 pathways, 8 drugs, 308 chemicals, and 3455 ingredients are included (Figure 2(a)). The DRDB server provides a user-friendly interface with five functional modules: search, prediction, compounds, target, and pathway. In addition, the help module contains the usage guidelines of DRDB. As illustrated in Figure 2(b), users can browse relevant entries by clicking corresponding sub-menus. For example, users can browse medications for DR therapy using the “Compounds” tool, as well as drugs against 15 proteins related to DR and ingredients of herbs for treating other diseases. On the “Search” screen, users can not only enter drug and protein names but also enter a specific structure. In addition, users can define query structure types, such as substructure search and similarity search in search interface.

The main characteristic of DRDB is its capacity to evaluate whether a given small molecule can target 15 DR-related targets. In prediction mode, users could select a specific target from the drop down list and upload a query molecule in smiles format. After about a half-minute of calculation, the prediction results of 16 models for each target will be displayed.  Figure 2(c) depicts the QSAR-based predicted results based on multiple binary classifiers with two types of chemical fingerprints—MACCS and ECFP6, and protein descriptors, and four algorithms—RF, SVM, KNN, and NN. Each classifies returns prediction result with the value of 0 or 1. If more than nine of 16 classifiers return 1, then this compound is more likely to be active against the corresponding target. With a large number of molecules, however, “Single Prediction” can become ineffective. In that situation, users can utilize the “Batch prediction” submenus to upload a file containing numerous molecules (maximum 1000) stored in sdf or SMILES formats, as well as enter a valid email address for obtaining calculation results. In a word, DRDB is designed to facilitate the identification of active compounds and target identification for the treatment of DR.

To evaluate the application of DRDB, the prediction of polypharmacology for CDDP was conducted as follow the case study.

### 3.3. Case Study: Systematic Analysis of the Multiple Bioactivities of CDDP

**Figure 3:**
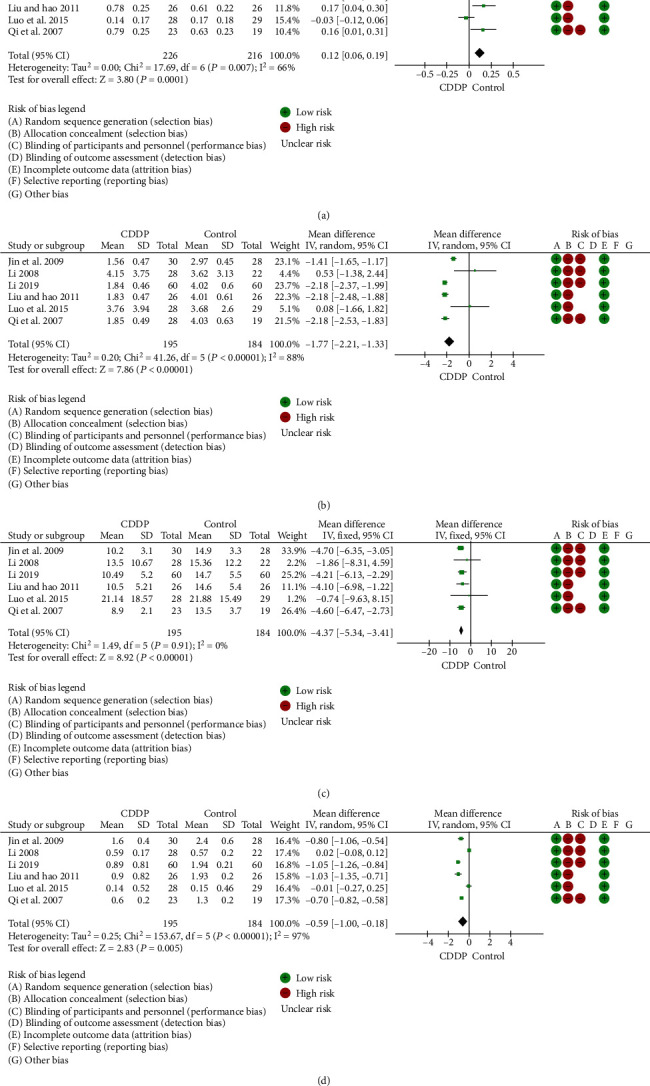
Meta-analysis of the effect of CDDP on patients with DR. (a) Vision. Seven studies provided visual acuity data with heterogeneity (*P* = 0.007, *I*^2^ = 66%), which was related to the intervention, observation methods, and duration of treatment, so a random effects model was used for analysis. The combined effect of seven studies was statistically significant (*P* < 0.01). (b) Gray value of visual field. Six studies were included in the analysis, and there was heterogeneity among the studies (*P* < 0.01, *I*^2^ = 88%). The combined effects of the six studies were statistically significant (*P* < 0.01) analyzed by a random effects model for combined analysis. (c) Microaneurysms. Six studies provided microaneurysm data and had no heterogeneity (*P* = 0.91, *I*^2^ = 0%). A fixed effects model was used for analysis. (d) Area of hemorrhagic focus. Six studies were included in the analysis, and there was heterogeneity among the studies (*P* < 0.01, *I*^2^ = 97%). A random effects model was used for combined analysis.

Compound Danshen Dripping Pills (CDDP) are a classic traditional Chinese medicine prescription, which is commonly used in the treatment of various cardiovascular diseases. Also, CDDP is being studied to treat DR. Thus, 15 studies were selected for the evaluation of CDDP effectiveness in alleviating DR-related symptoms. The information and quality of included studies were available as supplementary data (Figure S1, Table S2 and S3). The meta-analysis indicated that the curative effect of CDDP for DR was shown to be superior to controls, and this was significantly different for the improvements in vision, visual field, microaneurysms, and hemorrhage (Figures 3(a)–3(d)).

**Figure 4:**
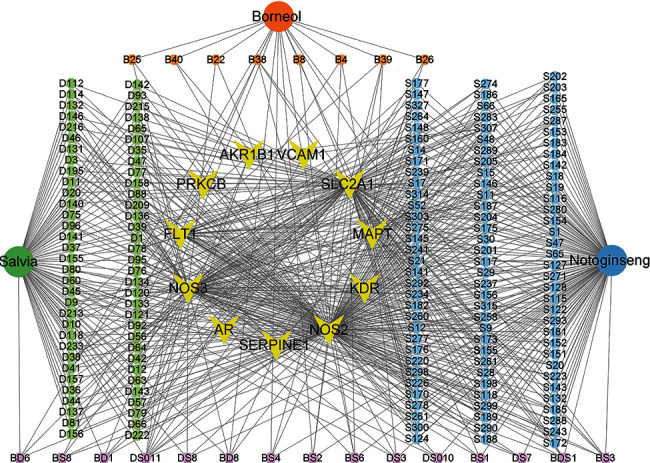
The compound-protein interaction network of Salvia miltiorrhiza, Panax notoginseng, and borneol based on DRDB. Triangle, ellipse, and circle represent protein nodes, herb nodes, and ingredient nodes, respectively.

Traditional Chinese medicine (TCM) exerts biological effects through interfering multiple biological targets by the synergic effects of many chemical components. To systematically analyze the action mechanisms of CDDP against DR, the potential targets of three main herbs in CDDP (Salvia miltiorrhiza, Panax notoginseng, and borneol) were predicted based on DRDB. The predicted associations between ingredients from three main herbs in CDDP and 15 target proteins are presented in Table S4. Based on the multiple voting method, the positive result from more than nine classifiers of each target is adopted for further analysis. The prediction results are integrated to construct a compound-protein interaction network. As shown in Figure 4, Salvia miltiorrhiza, Panax notoginseng, and borneol could target 15 targets from an overall perspective. The compound nodes with degree value that is more than the median degree (median degree = 1.8) of all nodes are retained. The degree analysis demonstrate that one compound could simultaneously interact with multiple targets, with an average two targets for one compound. Similarly, one target could interact with multiple molecules, with an average molecules of 46 for one target.

**Figure 5:**
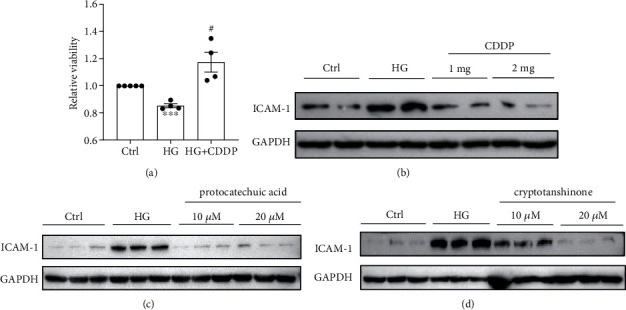
Target validation in ARPE19 cells. Ctrl: control group; HG: high glucose group. (a) The effect of CDDP on the relative viability of ARPE19 cells. (b) The effect of CDDP on the expression of ICAM-1 in the presence of high glucose. (c) The effect of protocatechuic acid on the expression of ICAM-1 in the presence of high glucose. (d) The effect of cryptotanshinone on the expression of ICAM-1 in the presence of high glucose.

The meta-analysis showed that CDDP could effectively treat DR, and the prediction results based on DRDB also showed that cryptotanshinone and protocatechuic acid in CDDP could interact with some targets associated with DR. In order to validate the corresponding relationship between the two components (cryptotanshinone and protocatechuic acid) and targets associated with DR, a hyperglucose cell model was constructed with the human RPE cell line ARPE-19 by high glucose stimulation and for target validation. As shown in Figure 5, high glucose significantly reduced ARPE-19 cell viability, which was improved by CDDP (Figure 5(a)). ICAM-1, one of the key targets involved in inflammation and acts as a local intensifying signal in the pathological processes associated with DR, was induced by the stimulation of high glucose, and this increase can be reversed by CDDP, as well as cryptotanshinone and protocatechuic acid, two key components of CDDP, which are consistent with our prediction based on QSAR models (Figures 5(b)–5(d)).

The complicated pathogenesis of DR may be associated with multiple proteins. In silico prediction of chemical-protein interactions (CPI) based on multitarget quantitative structure-activity relationship (mt-QSAR) method plays a vital role in target prediction and identification of optimal candidates in drug discovery of complicated disease. In this work, a total of 128 binary classifiers for 15 targets associated with DR were constructed to predict the CPIs. The results of 5-fold cross-validation and test set validation suggested that the classifiers have moderate classification performance. Generally, the limitations of machine learning algorithms, e.g., the type of molecular fingerprints and composition of training set, have a major impact on the accuracy of the classifiers. For example, the eight classifiers from MAPT (71 compounds in the training set) did the worst performance among the 15 targets in this study. Perhaps the main reason for this is due to few compounds included in the training set, which limits to a narrow application domain of classifiers target MAPT. With the advantage of based only on compounds structural information, machine learning methods in this study could be applied to predict other types of drug-related interactions. For example, there are evidence suggests that microRNA may affect gene expression and disease progression. More and more computational methods have been developed to identify the potential small molecule-miRNA associations and achieve good predictive performance [51, 52]. Machine learning methods in this study are expected to be used in identification of the potential small molecule-miRNA associations.

In general, the computational complexity of machine learning classifiers and the demand of particular operation system and software compiler limit the use of these models. To facilitate the application of multiple classifiers against 15 targets in drug discovery, 128 binary classifiers and chemogenomics information associated with DR were integrated into a free web server named DRDB, which included 15 genes, 157 pathways, 8 drugs, 308 chemicals, and 3455 ingredients. For case study, the applicability of DRDB was illustrated to systematically analyze the multiple bioactivities of CDDP against DR. The prediction results showed that one compound could simultaneously interact with multiple targets based on network pharmacology approach. Cryptotanshinone and protocatechuic acid, two key components of CDDP, could target ICAM-1 related to DR in vitro experiment. DRDB has potential applications towards target prediction and identification of optimal candidates and network pharmacology.

## 4. Conclusions

In this study, a chemogenomics database associated with DR was developed. The developed system provides implementation of 128 binary classifiers for the target identification and drug discovery for DR treatment. DRDB, a computational server, is available for discovery of multitarget ligands to combat DR and systematic prediction of CPIs based on mt-QSAR method. DRDB could contribute to systematically understand the pharmacological mechanisms of traditional Chinese medicine (TCM). In addition, the applicability of DRDB was illustrated through systematic analysis of multiple bioactivities of CDDP based on network pharmacology approach. In vitro experimental validation demonstrated that cryptotanshinone and protocatechuic acid, two key components of CDDP, could target ICAM-1 related to DR. These active compounds and CPIs could provide a basis for pharmacological profiles of CDDP therapy in DR. We hope that DRDB server could facilitate the discovery of new drugs and treatments for DR.

## Figures and Tables

**Table 1:** Performance summary of the 5-fold cross-validation for 15 targets towards DR.

Target	Fingerprint	Random Forest					KNN					SVM					Neural networks				
		AUC	SE	SP	*Q*	MCC	AUC	SE	SP	*Q*	MCC	AUC	SE	SP	*Q*	MCC	AUC	SE	SP	*Q*	MCC
ACE	MACCS	0.821	0.863	0.779	0.823	0.646	0.817	0.909	0.725	0.821	0.648	0.813	0.838	0.788	0.814	0.628	0.823	0.822	0.824	0.823	0.646
	ECFP6	0.808	0.855	0.761	0.810	0.620	0.809	0.822	0.797	0.810	0.619	0.816	0.871	0.761	0.819	0.638	0.797	0.846	0.748	0.799	0.598
AGTR1	MACCS	0.858	0.870	0.846	0.858	0.716	0.861	0.876	0.846	0.860	0.722	0.864	0.879	0.848	0.863	0.727	0.840	0.834	0.846	0.840	0.679
	ECFP6	0.879	0.885	0.873	0.878	0.757	0.876	0.910	0.843	0.876	0.754	0.879	0.899	0.859	0.878	0.758	0.862	0.870	0.854	0.862	0.724
AKR1B1	MACCS	0.716	0.736	0.696	0.715	0.432	0.719	0.696	0.743	0.720	0.439	0.734	0.733	0.736	0.734	0.468	0.714	0.692	0.736	0.715	0.429
	ECFP6	0.778	0.791	0.766	0.778	0.556	0.736	0.766	0.706	0.734	0.471	0.769	0.773	0.766	0.769	0.538	0.740	0.755	0.726	0.740	0.480
AR	MACCS	0.767	0.769	0.765	0.767	0.534	0.754	0.788	0.721	0.754	0.509	0.778	0.773	0.783	0.778	0.555	0.738	0.740	0.735	0.738	0.475
	ECFP6	0.767	0.788	0.747	0.767	0.535	0.763	0.805	0.721	0.762	0.527	0.768	0.798	0.739	0.768	0.537	0.756	0.766	0.747	0.756	0.512
FLT1	MACCS	0.770	0.778	0.762	0.770	0.540	0.753	0.755	0.751	0.753	0.506	0.770	0.769	0.771	0.770	0.540	0.741	0.735	0.748	0.741	0.483
	ECFP6	0.754	0.732	0.776	0.754	0.509	0.749	0.724	0.773	0.749	0.498	0.763	0.701	0.824	0.763	0.529	0.737	0.726	0.748	0.737	0.474
ICAM1	MACCS	0.754	0.797	0.710	0.754	0.509	0.739	0.812	0.667	0.739	0.483	0.775	0.855	0.696	0.775	0.558	0.783	0.812	0.754	0.783	0.566
	ECFP6	0.790	0.870	0.710	0.790	0.587	0.768	0.884	0.652	0.768	0.551	0.812	0.870	0.754	0.812	0.627	0.775	0.812	0.739	0.775	0.552
KDR	MACCS	0.787	0.801	0.773	0.787	0.574	0.777	0.791	0.763	0.777	0.554	0.800	0.800	0.800	0.800	0.600	0.762	0.775	0.748	0.762	0.524
	ECFP6	0.824	0.839	0.809	0.824	0.649	0.804	0.841	0.768	0.805	0.611	0.830	0.835	0.825	0.830	0.660	0.797	0.815	0.779	0.797	0.595
MAPT	MACCS	0.702	0.667	0.737	0.704	0.404	0.711	0.606	0.816	0.718	0.433	0.667	0.545	0.789	0.676	0.347	0.687	0.636	0.737	0.690	0.375
	ECFP6	0.783	0.697	0.868	0.789	0.577	0.770	0.697	0.842	0.775	0.547	0.743	0.697	0.789	0.746	0.489	0.754	0.667	0.842	0.761	0.519
NOS2	MACCS	0.812	0.818	0.807	0.812	0.624	0.830	0.825	0.834	0.830	0.659	0.820	0.847	0.793	0.819	0.640	0.794	0.796	0.793	0.794	0.589
	ECFP6	0.823	0.832	0.814	0.823	0.646	0.817	0.883	0.752	0.816	0.639	0.830	0.832	0.828	0.830	0.660	0.815	0.803	0.828	0.816	0.631
NOS3	MACCS	0.720	0.710	0.730	0.720	0.440	0.714	0.718	0.710	0.714	0.427	0.734	0.714	0.755	0.734	0.469	0.730	0.710	0.751	0.730	0.461
	ECFP6	0.741	0.763	0.718	0.741	0.482	0.718	0.788	0.647	0.718	0.440	0.745	0.776	0.714	0.745	0.491	0.726	0.730	0.722	0.726	0.452
PRKCB	MACCS	0.785	0.791	0.780	0.785	0.569	0.784	0.822	0.747	0.781	0.567	0.805	0.744	0.867	0.810	0.618	0.791	0.783	0.800	0.792	0.582
	ECFP6	0.828	0.829	0.827	0.828	0.655	0.816	0.891	0.740	0.810	0.633	0.839	0.845	0.833	0.839	0.677	0.823	0.806	0.840	0.824	0.647
SERPINE1	MACCS	0.801	0.819	0.783	0.800	0.601	0.780	0.810	0.750	0.778	0.558	0.820	0.848	0.792	0.818	0.638	0.804	0.800	0.808	0.804	0.608
	ECFP6	0.800	0.800	0.800	0.800	0.599	0.792	0.810	0.775	0.791	0.583	0.819	0.838	0.800	0.818	0.637	0.811	0.838	0.783	0.809	0.620
SLC2A1	MACCS	0.707	0.741	0.673	0.706	0.415	0.685	0.689	0.681	0.685	0.370	0.723	0.753	0.692	0.722	0.446	0.695	0.697	0.692	0.695	0.389
	ECFP6	0.733	0.797	0.669	0.732	0.469	0.731	0.769	0.692	0.730	0.462	0.746	0.769	0.723	0.746	0.492	0.736	0.765	0.708	0.736	0.473
TNF	MACCS	0.832	0.827	0.836	0.831	0.663	0.824	0.842	0.806	0.824	0.648	0.848	0.886	0.810	0.848	0.698	0.817	0.820	0.813	0.817	0.633
	ECFP6	0.859	0.846	0.873	0.859	0.719	0.863	0.875	0.851	0.863	0.726	0.859	0.879	0.840	0.859	0.719	0.842	0.864	0.821	0.843	0.686
VCAM1	MACCS	0.774	0.595	0.953	0.787	0.596	0.813	0.649	0.977	0.825	0.673	0.786	0.595	0.977	0.800	0.629	0.679	0.568	0.791	0.688	0.369
	ECFP6	0.788	0.622	0.953	0.800	0.619	0.799	0.622	0.977	0.812	0.651	0.813	0.649	0.977	0.825	0.673	0.735	0.703	0.767	0.738	0.471
PCM	MACCS_pro	0.776	0.784	0.767	0.776	0.552	0.769	0.783	0.755	0.769	0.539	0.787	0.776	0.797	0.787	0.573	0.75	0.743	0.757	0.75	0.501
	ECFP6_pro	0.810	0.821	0.799	0.810	0.619	0.788	0.832	0.743	0.788	0.578	0.805	0.810	0.800	0.805	0.610	0.781	0.800	0.762	0.781	0.562

**Table 2:** Performance summary of the test set external validation for 15 targets towards DR.

Target	Fingerprint	Random Forest					KNN					SVM					Neural networks				
		AUC	SE	SP	*Q*	MCC	AUC	SE	SP	*Q*	MCC	AUC	SE	SP	*Q*	MCC	AUC	SE	SP	*Q*	MCC
ACE	MACCS	0.806	0.885	0.727	0.810	0.623	0.744	0.869	0.618	0.750	0.506	0.799	0.852	0.745	0.802	0.603	0.743	0.885	0.600	0.750	0.510
	ECFP6	0.814	0.902	0.727	0.819	0.642	0.790	0.852	0.727	0.793	0.586	0.842	0.902	0.782	0.845	0.691	0.784	0.787	0.782	0.784	0.568
AGTR1	MACCS	0.884	0.888	0.880	0.884	0.768	0.851	0.843	0.859	0.851	0.702	0.868	0.876	0.859	0.867	0.735	0.872	0.843	0.902	0.873	0.747
	ECFP6	0.862	0.876	0.848	0.862	0.724	0.885	0.921	0.848	0.884	0.770	0.863	0.910	0.815	0.862	0.728	0.856	0.865	0.848	0.856	0.713
AKR1B1	MACCS	0.746	0.768	0.724	0.745	0.491	0.660	0.623	0.697	0.662	0.321	0.745	0.754	0.737	0.745	0.490	0.718	0.739	0.697	0.717	0.436
	ECFP6	0.741	0.797	0.684	0.738	0.483	0.734	0.797	0.671	0.731	0.470	0.753	0.783	0.724	0.752	0.506	0.734	0.797	0.671	0.731	0.470
AR	MACCS	0.754	0.788	0.721	0.753	0.509	0.762	0.849	0.675	0.760	0.531	0.794	0.836	0.753	0.793	0.590	0.758	0.815	0.701	0.757	0.519
	ECFP6	0.824	0.856	0.792	0.823	0.649	0.811	0.856	0.766	0.810	0.624	0.831	0.863	0.799	0.830	0.662	0.769	0.726	0.812	0.770	0.540
FLT1	MACCS	0.791	0.773	0.809	0.791	0.582	0.757	0.693	0.820	0.757	0.518	0.791	0.761	0.820	0.791	0.583	0.729	0.693	0.764	0.729	0.458
	ECFP6	0.808	0.750	0.865	0.808	0.620	0.751	0.705	0.798	0.751	0.505	0.802	0.705	0.899	0.802	0.616	0.780	0.773	0.787	0.780	0.559
ICAM1	MACCS	0.712	0.778	0.647	0.714	0.429	0.855	0.944	0.765	0.857	0.723	0.797	0.889	0.706	0.800	0.607	0.799	0.833	0.765	0.800	0.600
	ECFP6	0.858	0.833	0.882	0.857	0.716	0.858	0.833	0.882	0.857	0.716	0.858	0.833	0.882	0.857	0.716	0.971	1.000	0.941	0.971	0.944
KDR	MACCS	0.788	0.812	0.764	0.788	0.576	0.765	0.800	0.730	0.765	0.531	0.799	0.821	0.777	0.799	0.599	0.756	0.816	0.696	0.757	0.516
	ECFP6	0.829	0.827	0.831	0.829	0.658	0.812	0.837	0.788	0.813	0.625	0.833	0.838	0.828	0.833	0.666	0.805	0.830	0.780	0.805	0.611
MAPT	MACCS	0.838	0.875	0.800	0.833	0.671	0.838	0.875	0.800	0.833	0.671	0.750	1.000	0.500	0.722	0.555	0.900	1.000	0.800	0.889	0.800
	ECFP6	0.750	1.000	0.500	0.722	0.555	0.750	1.000	0.500	0.722	0.555	0.750	1.000	0.500	0.722	0.555	0.900	1.000	0.800	0.889	0.800
NOS2	MACCS	0.762	0.829	0.694	0.761	0.527	0.775	0.800	0.750	0.775	0.550	0.832	0.886	0.778	0.831	0.667	0.762	0.829	0.694	0.761	0.527
	ECFP6	0.832	0.886	0.778	0.831	0.667	0.790	0.914	0.667	0.789	0.598	0.762	0.829	0.694	0.761	0.527	0.704	0.714	0.694	0.704	0.409
NOS3	MACCS	0.719	0.721	0.717	0.719	0.438	0.686	0.689	0.683	0.686	0.372	0.686	0.689	0.683	0.686	0.372	0.678	0.639	0.717	0.678	0.357
	ECFP6	0.817	0.902	0.733	0.818	0.645	0.792	0.902	0.683	0.793	0.600	0.826	0.869	0.783	0.826	0.655	0.818	0.836	0.800	0.818	0.637
PRKCB	MACCS	0.783	0.750	0.816	0.786	0.567	0.825	0.781	0.868	0.829	0.654	0.838	0.781	0.895	0.843	0.684	0.764	0.844	0.684	0.757	0.529
	ECFP6	0.882	0.844	0.921	0.886	0.770	0.803	0.844	0.763	0.800	0.605	0.869	0.844	0.895	0.871	0.741	0.869	0.844	0.895	0.871	0.741
SERPINE1	MACCS	0.762	0.846	0.677	0.754	0.526	0.778	0.846	0.710	0.772	0.556	0.746	0.846	0.645	0.737	0.496	0.762	0.846	0.677	0.754	0.526
	ECFP6	0.704	0.731	0.677	0.702	0.407	0.749	0.692	0.806	0.754	0.503	0.813	0.885	0.742	0.807	0.627	0.813	0.885	0.742	0.807	0.627
SLC2A1	MACCS	0.789	0.810	0.769	0.789	0.579	0.719	0.714	0.723	0.719	0.437	0.758	0.778	0.738	0.758	0.516	0.720	0.778	0.662	0.719	0.442
	ECFP6	0.704	0.778	0.631	0.703	0.413	0.711	0.746	0.677	0.711	0.424	0.727	0.762	0.692	0.727	0.455	0.688	0.730	0.646	0.688	0.377
TNF	MACCS	0.890	0.899	0.881	0.890	0.779	0.875	0.870	0.881	0.875	0.750	0.860	0.899	0.821	0.860	0.722	0.838	0.855	0.821	0.838	0.677
	ECFP6	0.889	0.928	0.851	0.890	0.781	0.889	0.913	0.866	0.890	0.780	0.874	0.913	0.836	0.875	0.752	0.845	0.855	0.836	0.846	0.691
VCAM1	MACCS	0.742	0.667	0.818	0.750	0.492	0.833	0.667	1.000	0.850	0.724	0.778	0.556	1.000	0.800	0.638	0.596	0.556	0.636	0.600	0.192
	ECFP6	0.732	0.556	0.909	0.750	0.504	0.778	0.556	1.000	0.800	0.638	0.778	0.556	1.000	0.800	0.638	0.732	0.556	0.909	0.750	0.504
PCM	MACCS_pro	0.788	0.808	0.769	0.788	0.577	0.77	0.8	0.741	0.77	0.542	0.799	0.8	0.798	0.799	0.597	0.758	0.726	0.79	0.758	0.518
	ECFP6_pro	0.821	0.836	0.806	0.821	0.643	0.803	0.842	0.764	0.803	0.608	0.818	0.823	0.813	0.818	0.636	0.795	0.814	0.775	0.795	0.59

**Table 3:** Performance summary of the multivoting ensemble method on integrated test set.

Cutoff	AUC	*Q*	SE	SP	MCC
1	0.724	0.724	0.957	0.491	0.507
2	0.766	0.766	0.936	0.596	0.566
3	0.789	0.789	0.916	0.663	0.597
4	0.802	0.802	0.903	0.701	0.617
5	0.810	0.810	0.888	0.731	0.627
6	0.815	0.815	0.879	0.751	0.635
7	0.817	0.817	0.863	0.770	0.636
8	0.821	0.821	0.843	0.799	0.643
9	0.824	0.824	0.820	0.828	0.648
10	0.819	0.819	0.795	0.844	0.64
11	0.816	0.816	0.771	0.86	0.634
12	0.814	0.814	0.749	0.879	0.633
13	0.803	0.803	0.709	0.897	0.617
14	0.792	0.792	0.665	0.919	0.603
15	0.765	0.766	0.591	0.940	0.566
16	0.686	0.686	0.409	0.962	0.446

## Data Availability

The data used to support the findings of this study are included within the article and supplementary information files.

## Supplementary Materials

Supplementary Materials The supplementary description includes meta-analysis, the predicted associations between ingredients from three main herbs in CDDP and 15 target proteins, and the molecular data set in the ChEMBL database of each gene selected for model construction.
