# Supplementary material for: DRDB: A Machine Learning Platform to Predict Chemical-Protein Interactions towards Diabetic Retinopathy
Source: Oxid Med Cell Longev. 2022 Jul 20;2022:1718353. doi: 10.1155/2022/1718353 (PMC9329024; doi:10.1155/2022/1718353)
Supplement: Supplementary Materials — The supplementary description includes meta-analysis, the predicted associations between ingredients from three main herbs in CDDP and 15 target proteins, and the molecular data set in the ChEMBL database of each gene selected for model construction. [file 1718353.f1.docx]

# DRDB: A machine learning platform to predict chemical-protein interactions towards diabetic retinopathy

Yu Wei^1,#^, Ruili Zhang^1,#^, Xiaoqiang Li^2,#^, Zhonglin Li^1,#^, Kaimin Guo^2^, Shanshan Li^1^, Li Yan^1^, Qian Zhao^2^, Baijian Qu^1^, Wenjia Wang^2^, Shuiping Zhou^3^, He Sun^2,3^, Jianping Lin^1,*^, Yunhui Hu^2,*^

^1^ State Key Laboratory of Medicinal Chemical Biology, College of Pharmacy and Tianjin Key Laboratory of Molecular Drug Research, Nankai University, Haihe Education Park, 38 Tongyan Road, Tianjin, 300353, China
^2^ Cloudphar Pharmaceuticals Co., Ltd., Shenzhen, China
^3^ The State Key Laboratory of Core Technology in Innovative Chinese Medicine, Tasly Academy,Tasly Holding Group Co.,Ltd, No. 1, Tingjiang West Road, Beichen District, Tianjin, 300410,China

Correspondence: jianpinglin@nankai.edu.cn (J. Lin) tsl-huyunhui@tasly.com (Y. Hu)

# ^#^ These authors contribute equally.

**Table S1** The molecular data set in the ChEMBL database of each gene selected for model construction.

| **Gene** | **Active molecules** | **Inactive molecules** | **Total** |
| --- | --- | --- | --- |
| ACE | 302(pIC50≥6.5) | 277(pIC50<6.5) | 579 |
| AGTR1 | 444(pIC50≥7.5) | 461(pIC50<7.5) | 905 |
| FLT1 | 439(pIC50≥6.5) | 442(pIC50<6.5) | 881 |
| PRKCB | 161(pIC50≥7.0) | 188(pIC50<7.0) | 349 |
| AKR1B1 | 342(pIC50≥6.5) | 379(pIC50<6.5) | 721 |
| AR | 731(pIC50≥6.6) | 766(pIC50<6.6) | 1497 |
| ICAM1 | 87(pIC50≥6.6) | 86(pIC50<6.6) | 173 |
| MAPT | 41(pIC50≥6.2) | 48(pIC50<6.2) | 89 |
| NOS2 | 172(pIC50≥5.0) | 181(pIC50<5.0) | 353 |
| NOS3 | 302(pIC50≥4.8) | 301(pIC50<4.8) | 603 |
| SERPINE1 | 131(pIC50≥5.5) | 151(pIC50<5.5) | 282 |
| SLC2A1 | 314(pIC50≥5.3) | 325(pIC50<5.3) | 639 |
| TNF | 341(pIC50≥5.8) | 335(pIC50<5.8) | 676 |
| VCAM1 | 46(pIC50≥5.2) | 54(pIC50<5.2) | 100 |
| KDR | 3823(pIC50≥6.8) | 3699(pIC50<6.8) | 7522 |

***Meta analysis***

*Inclusion criteria and exclusion criteria*

Inclusion criteria were the following: a) randomized controlled trial; b) the selected patients were diagnosed with diabetic retinopathy; c) Compound Danshen Dripping Pills was taken as the main intervention in the treatment group compared to the control group; d) the selected literature are comparable, which have similar study methods, comprehensive statistical indicators and completed general information.

Exclusion criteria were the following: a) repeating publications; b) Non-randomized clinical controlled study; c)Retrospective study; d) Animal experimental studies; f) Combination with other drugs or methods.

*Data Extraction*

The following data were collected from each study: author, publication date, sample size of cases, age, intervention, duration of treatment, vision, gray value of visual field, microaneurysm changes, area of hemorrhagic focus, and adverse effects.

*Study quality assessment*

The Cochrane Systematic Review Manual is used to assess the study quality.The evaluation criteria include randomization, allocation concealment, degrees of blinding, blinding of result assessment, incomplete result data, selective reporting, and other biases.

***Information of included studies***

A total of 264 studies were identified, and 15 studies were included after selecting. The studies selection process and results are shown in Supplementary materials Figure S1. In these 15 studies, there were 519 and 503 people in the intervention group and the control group, respectively. All the participants were DR patients, who were diagnosed by the authoritative criteria of DR. In the intervention group, CDDP was used as an intervention. While, calcium dobesilate was used in 11 studies of the control group. In addition, vitamin B and / or vitamin C and /or LuDing tablets and/or PanShengDing tablets and /or inosine tablets, Pancreatic kininogen were used in 4 studies. The follow-up period ranged from 2 to 6 months, and the selected studies are summarized in Supplementary materials Table S2.

***Quality of included studies***

All of the 15 included studies referred to randomized controlled trials, but did not explicitly describe the method and blinding of random sequence generation. All the studies showed that the two groups of data were comparable, and no cases of withdrawal and loss to follow-up were recorded. No adverse events were recorded in three studies. Methodological quality was assessed as high risk.

**Supplementary materials**

Records after duplicates were removed

(n = 155)

Records screened again

(n = 86)

Studies assessed for eligibility

(n = 15)

Meeting the exclusion criteria (n = 58)

Not meeting the inclusion criteria (n = 13)

Studies found in initial research (n = 264)

CNKI (n = 97)

WanFang (n = 94)

VIP (n = 66)

PubMed (n = 7)

Retrospective study (n = 5)

Animal studies (n = 1)

Non-controlled clinical trials (n= 1)

Not related to subject (n = 16)

**Figure S1**. The flow chart of studies selection process.

**Table S2**. The information of studies.

| **Studies** | **Sample**  **(E/C)** | **Age (E/C) (year)** | **Intervention** | **Contol** | **Duration**  **(months)** | **Outcomes measured** |
| --- | --- | --- | --- | --- | --- | --- |
| Li 2019 | 60 / 60 | 58.11 ± 3.43 / 58.00 ± 3.56 | CDDP | Calcium dobesilate | 4 months | 1. ③④ |
| Wang et al. 2016 | 45 / 45 | 47-77 / 48-76 | CDDP | Calcium dobesilate | 2 months | ①⑤ |
| Liu 2016 | 24 / 24 | 30-70 | CDDP | Calcium dobesilate | 6 months | 1. ④⑤ |
| Luo et al. 2015 | 28 / 29 | 59.54 ± 7.46 / 57.86 ±1 0.03 | CDDP | Calcium dobesilate | 3 months | ①③④ |
| Chen 2013 | 43 / 43 | 52.6 ± 3.5 | CDDP | Calcium dobesilate | 6 months | ③④ |
| Peng 2012 | 43 / 43 | 21-67 | CDDP | Calcium dobesilate | 6 months | ③④ |
| Xu 2011 | 40 / 40 | 52.3 / 54.5 | CDDP | LuDing tablets + vitamin C  + PanShengDing tablets | 3 months | ①② |
| Liu and Hao 2011 | 26 / 26 | 39-76 | CDDP | Vitamin B + vitamin C + Inosine Tablets | 3 months | ①②③④ |
| Li and Chen 2011 | 31 / 32 | 54.60 ± 10.40 / 58.12 ± 9.31 | CDDP | Calcium dobesilate | 3 months | ①⑤ |
| Jin et al. 2009 | 30 / 28 | 62.78 ± 7.69 / 61.11 ± 7.27 | CDDP | Calcium dobesilate | 3 months | ①②③④ |
| Li 2008 | 28 / 22 | 60.00 ± 8.83 / 60.88 ± 7.5 | CDDP | Calcium dobesilate | 3 months | ①②⑤ |
| Qi et al. 2007 | 23 / 19 | 36-72 | CDDP | Vitamin B1 + LuDing  tablets | 3 months | ①②③④ |
| He and Cai 2007 | 30 / 30 | 58.78 ± 6.78 / 59.01 ± 6.62 | CDDP | Pancreatic kininogen | 3 months | ①⑤ |
| Chen and Zhong 2006 | 31 / 32 | 54.60 ± 10.40 / 58.12 ± 9.31 | CDDP | Calcium dobesilate | 3 months | ①⑤ |
| Bao et al. 2006 | 37 / 30 | 58.19 / 57.20 | CDDP | Calcium dobesilate | 6 months | ③④ |

Note: ①vision, ②gray value of visual field, ③microaneurysm, ④hemorrhage, ⑤FFA

**Table S3**. Quality assessment of studies.

| **Studies** | | **Adequate sequence generation** | **Allocation concealment** | **Blinding of participants and personnel** | **Binding of outcome assessment** | **Incomplete outcome** | **Selective reporting** | **other biases** |
| --- | --- | --- | --- | --- | --- | --- | --- | --- |
| Li 2019 | | Low | High | High | unclear | Low | unclear | unclear |
| Wang et al. 2016 | | Low | High | High | unclear | Low | unclear | unclear |
| Liu 2016 | | Low | High | High | unclear | Low | unclear | unclear |
| Luo et al. 2015 | | Low | High | unclear | unclear | Low | unclear | unclear |
| Chen 2013 | | Low | High | unclear | unclear | Low | unclear | unclear |
| Peng 2012 | | Low | High | unclear | unclear | Low | unclear | unclear |
| Xu 2011 | Low | | High | unclear | unclear | Low | unclear | unclear |
| Liu and Hao 2011 | Low | | High | unclear | unclear | Low | unclear | unclear |
| Li and Chen 2011 | Low | | High | unclear | unclear | Low | unclear | unclear |
| Jin et al. 2009 | Low | | High | unclear | unclear | Low | unclear | unclear |
| Li 2008 | Low | | High | High | unclear | Low | unclear | unclear |
| Qi et al. 2007 | Low | | High | High | unclear | Low | unclear | unclear |
| He and Cai 2007 | Low | | High | unclear | unclear | Low | unclear | unclear |
| Chen and Zhong 2006 | Low | | High | unclear | unclear | Low | unclear | unclear |
| Bao et al. 2006 | Low | | High | unclear | unclear | Low | unclear | unclear |

**Table S4**. The predicted associations between ingredients from three main herbs in CDDP and 15 target proteins.

| Molecule | Number of classifiers | Target | Source |
| --- | --- | --- | --- |
| D213 | 15 | AKR1B1 | Salvia |
| D215 | 10 | AKR1B1 | Salvia |
| D222 | 12 | AKR1B1 | Salvia |
| DS011 | 9 | AKR1B1 | Salvia/Notoginseng |
| S307 | 11 | AKR1B1 | Notoginseng |
| S314 | 12 | AKR1B1 | Notoginseng |
| S17 | 9 | AR | Notoginseng |
| S181 | 9 | AR | Notoginseng |
| S185 | 9 | AR | Notoginseng |
| S201 | 9 | AR | Notoginseng |
| S205 | 9 | AR | Notoginseng |
| S298 | 9 | AR | Notoginseng |
| B38 | 10 | FLT1 | Borneol |
| B39 | 10 | FLT1 | Borneol |
| D120 | 11 | FLT1 | Salvia |
| D121 | 11 | FLT1 | Salvia |
| D138 | 9 | FLT1 | Salvia |
| D195 | 10 | FLT1 | Salvia |
| D216 | 9 | FLT1 | Salvia |
| DS011 | 13 | FLT1 | Salvia/Notoginseng |
| S127 | 9 | FLT1 | Notoginseng |
| S173 | 9 | FLT1 | Notoginseng |
| S175 | 11 | FLT1 | Notoginseng |
| S176 | 12 | FLT1 | Notoginseng |
| S181 | 10 | FLT1 | Notoginseng |
| S182 | 12 | FLT1 | Notoginseng |
| S183 | 11 | FLT1 | Notoginseng |
| S184 | 12 | FLT1 | Notoginseng |
| S185 | 11 | FLT1 | Notoginseng |
| S187 | 11 | FLT1 | Notoginseng |
| S188 | 11 | FLT1 | Notoginseng |
| S189 | 12 | FLT1 | Notoginseng |
| S20 | 10 | FLT1 | Notoginseng |
| S201 | 10 | FLT1 | Notoginseng |
| S202 | 12 | FLT1 | Notoginseng |
| S203 | 11 | FLT1 | Notoginseng |
| S204 | 12 | FLT1 | Notoginseng |
| S205 | 11 | FLT1 | Notoginseng |
| S220 | 9 | FLT1 | Notoginseng |
| S264 | 9 | FLT1 | Notoginseng |
| S269 | 10 | FLT1 | Notoginseng |
| S271 | 10 | FLT1 | Notoginseng |
| S280 | 9 | FLT1 | Notoginseng |
| S281 | 10 | FLT1 | Notoginseng |
| S283 | 9 | FLT1 | Notoginseng |
| S298 | 10 | FLT1 | Notoginseng |
| S299 | 10 | FLT1 | Notoginseng |
| S303 | 10 | FLT1 | Notoginseng |
| S307 | 16 | FLT1 | Notoginseng |
| DS011 | 11 | ICAM1 | Salvia/Notoginseng |
| B40 | 9 | KDR | Borneol |
| D114 | 9 | KDR | Salvia |
| D118 | 13 | KDR | Salvia |
| D120 | 11 | KDR | Salvia |
| D121 | 11 | KDR | Salvia |
| DS011 | 9 | KDR | Salvia/Notoginseng |
| S298 | 9 | KDR | Notoginseng |
| S307 | 12 | KDR | Notoginseng |
| B38 | 11 | MAPT | Borneol |
| B39 | 11 | MAPT | Borneol |
| B4 | 12 | MAPT | Borneol |
| BD1 | 9 | MAPT | Borneol/Salvia |
| BDS1 | 9 | MAPT | Borneol/Salvia/Notoginseng |
| BS3 | 10 | MAPT | Borneol/Notoginseng |
| D110 | 9 | MAPT | Salvia |
| D38 | 10 | MAPT | Salvia |
| D41 | 9 | MAPT | Salvia |
| D9 | 10 | MAPT | Salvia |
| DS011 | 13 | MAPT | Salvia/Notoginseng |
| DS3 | 10 | MAPT | Salvia/Notoginseng |
| S124 | 10 | MAPT | Notoginseng |
| S141 | 9 | MAPT | Notoginseng |
| S142 | 9 | MAPT | Notoginseng |
| S143 | 10 | MAPT | Notoginseng |
| S146 | 10 | MAPT | Notoginseng |
| S18 | 10 | MAPT | Notoginseng |
| S181 | 10 | MAPT | Notoginseng |
| S182 | 10 | MAPT | Notoginseng |
| S185 | 9 | MAPT | Notoginseng |
| S19 | 9 | MAPT | Notoginseng |
| S201 | 10 | MAPT | Notoginseng |
| S202 | 10 | MAPT | Notoginseng |
| S205 | 9 | MAPT | Notoginseng |
| S264 | 10 | MAPT | Notoginseng |
| S287 | 9 | MAPT | Notoginseng |
| S29 | 9 | MAPT | Notoginseng |
| S30 | 9 | MAPT | Notoginseng |
| B10 | 9 | NOS2 | Borneol |
| B12 | 10 | NOS2 | Borneol |
| B2 | 9 | NOS2 | Borneol |
| B22 | 11 | NOS2 | Borneol |
| B25 | 9 | NOS2 | Borneol |
| B26 | 10 | NOS2 | Borneol |
| B3 | 9 | NOS2 | Borneol |
| B31 | 14 | NOS2 | Borneol |
| B32 | 15 | NOS2 | Borneol |
| B33 | 12 | NOS2 | Borneol |
| B38 | 10 | NOS2 | Borneol |
| B39 | 10 | NOS2 | Borneol |
| B4 | 9 | NOS2 | Borneol |
| B40 | 9 | NOS2 | Borneol |
| B6 | 12 | NOS2 | Borneol |
| B7 | 11 | NOS2 | Borneol |
| B8 | 13 | NOS2 | Borneol |
| B9 | 9 | NOS2 | Borneol |
| BD1 | 12 | NOS2 | Borneol/Salvia |
| BD2 | 11 | NOS2 | Borneol/Salvia |
| BD3 | 9 | NOS2 | Borneol/Salvia |
| BD4 | 10 | NOS2 | Borneol/Salvia |
| BD5 | 11 | NOS2 | Borneol/Salvia |
| BD6 | 11 | NOS2 | Borneol/Salvia |
| BD8 | 10 | NOS2 | Borneol/Salvia |
| BDS1 | 10 | NOS2 | Borneol/Salvia/Notoginseng |
| BDS2 | 12 | NOS2 | Borneol/Salvia/Notoginseng |
| BDS7 | 11 | NOS2 | Borneol/Salvia/Notoginseng |
| BS1 | 11 | NOS2 | Borneol/Notoginseng |
| BS2 | 13 | NOS2 | Borneol/Notoginseng |
| BS3 | 10 | NOS2 | Borneol/Notoginseng |
| BS4 | 13 | NOS2 | Borneol/Notoginseng |
| BS5 | 10 | NOS2 | Borneol/Notoginseng |
| BS6 | 14 | NOS2 | Borneol/Notoginseng |
| BS7 | 10 | NOS2 | Borneol/Notoginseng |
| BS8 | 9 | NOS2 | Borneol/Notoginseng |
| D1 | 10 | NOS2 | Salvia |
| D10 | 14 | NOS2 | Salvia |
| D103 | 10 | NOS2 | Salvia |
| D105 | 10 | NOS2 | Salvia |
| D107 | 14 | NOS2 | Salvia |
| D11 | 16 | NOS2 | Salvia |
| D111 | 10 | NOS2 | Salvia |
| D112 | 13 | NOS2 | Salvia |
| D114 | 12 | NOS2 | Salvia |
| D115 | 13 | NOS2 | Salvia |
| D116 | 10 | NOS2 | Salvia |
| D118 | 11 | NOS2 | Salvia |
| D12 | 13 | NOS2 | Salvia |
| D120 | 15 | NOS2 | Salvia |
| D121 | 15 | NOS2 | Salvia |
| D122 | 9 | NOS2 | Salvia |
| D125 | 12 | NOS2 | Salvia |
| D126 | 11 | NOS2 | Salvia |
| D127 | 9 | NOS2 | Salvia |
| D128 | 14 | NOS2 | Salvia |
| D129 | 12 | NOS2 | Salvia |
| D13 | 13 | NOS2 | Salvia |
| D130 | 11 | NOS2 | Salvia |
| D131 | 10 | NOS2 | Salvia |
| D132 | 14 | NOS2 | Salvia |
| D133 | 9 | NOS2 | Salvia |
| D134 | 11 | NOS2 | Salvia |
| D136 | 14 | NOS2 | Salvia |
| D137 | 14 | NOS2 | Salvia |
| D138 | 11 | NOS2 | Salvia |
| D14 | 10 | NOS2 | Salvia |
| D140 | 10 | NOS2 | Salvia |
| D141 | 12 | NOS2 | Salvia |
| D142 | 14 | NOS2 | Salvia |
| D143 | 11 | NOS2 | Salvia |
| D145 | 11 | NOS2 | Salvia |
| D146 | 14 | NOS2 | Salvia |
| D147 | 13 | NOS2 | Salvia |
| D148 | 9 | NOS2 | Salvia |
| D150 | 11 | NOS2 | Salvia |
| D151 | 13 | NOS2 | Salvia |
| D153 | 11 | NOS2 | Salvia |
| D155 | 11 | NOS2 | Salvia |
| D156 | 12 | NOS2 | Salvia |
| D157 | 11 | NOS2 | Salvia |
| D158 | 11 | NOS2 | Salvia |
| D159 | 9 | NOS2 | Salvia |
| D16 | 13 | NOS2 | Salvia |
| D17 | 9 | NOS2 | Salvia |
| D19 | 12 | NOS2 | Salvia |
| D193 | 11 | NOS2 | Salvia |
| D194 | 13 | NOS2 | Salvia |
| D195 | 11 | NOS2 | Salvia |
| D199 | 10 | NOS2 | Salvia |
| D2 | 15 | NOS2 | Salvia |
| D20 | 14 | NOS2 | Salvia |
| D202 | 11 | NOS2 | Salvia |
| D209 | 10 | NOS2 | Salvia |
| D210 | 11 | NOS2 | Salvia |
| D212 | 10 | NOS2 | Salvia |
| D213 | 14 | NOS2 | Salvia |
| D222 | 10 | NOS2 | Salvia |
| D23 | 9 | NOS2 | Salvia |
| D232 | 9 | NOS2 | Salvia |
| D233 | 12 | NOS2 | Salvia |
| D24 | 12 | NOS2 | Salvia |
| D25 | 11 | NOS2 | Salvia |
| D27 | 12 | NOS2 | Salvia |
| D28 | 12 | NOS2 | Salvia |
| D29 | 9 | NOS2 | Salvia |
| D3 | 15 | NOS2 | Salvia |
| D30 | 11 | NOS2 | Salvia |
| D31 | 11 | NOS2 | Salvia |
| D32 | 14 | NOS2 | Salvia |
| D35 | 12 | NOS2 | Salvia |
| D36 | 12 | NOS2 | Salvia |
| D37 | 14 | NOS2 | Salvia |
| D38 | 14 | NOS2 | Salvia |
| D39 | 16 | NOS2 | Salvia |
| D41 | 12 | NOS2 | Salvia |
| D42 | 13 | NOS2 | Salvia |
| D43 | 15 | NOS2 | Salvia |
| D44 | 11 | NOS2 | Salvia |
| D45 | 14 | NOS2 | Salvia |
| D46 | 12 | NOS2 | Salvia |
| D47 | 14 | NOS2 | Salvia |
| D48 | 12 | NOS2 | Salvia |
| D49 | 9 | NOS2 | Salvia |
| D5 | 9 | NOS2 | Salvia |
| D50 | 12 | NOS2 | Salvia |
| D51 | 10 | NOS2 | Salvia |
| D55 | 9 | NOS2 | Salvia |
| D56 | 12 | NOS2 | Salvia |
| D57 | 16 | NOS2 | Salvia |
| D58 | 12 | NOS2 | Salvia |
| D59 | 12 | NOS2 | Salvia |
| D6 | 11 | NOS2 | Salvia |
| D60 | 15 | NOS2 | Salvia |
| D61 | 9 | NOS2 | Salvia |
| D63 | 11 | NOS2 | Salvia |
| D64 | 10 | NOS2 | Salvia |
| D65 | 12 | NOS2 | Salvia |
| D66 | 13 | NOS2 | Salvia |
| D67 | 10 | NOS2 | Salvia |
| D7 | 14 | NOS2 | Salvia |
| D74 | 10 | NOS2 | Salvia |
| D75 | 9 | NOS2 | Salvia |
| D76 | 14 | NOS2 | Salvia |
| D77 | 13 | NOS2 | Salvia |
| D78 | 14 | NOS2 | Salvia |
| D79 | 15 | NOS2 | Salvia |
| D8 | 12 | NOS2 | Salvia |
| D80 | 13 | NOS2 | Salvia |
| D81 | 12 | NOS2 | Salvia |
| D83 | 12 | NOS2 | Salvia |
| D85 | 11 | NOS2 | Salvia |
| D87 | 12 | NOS2 | Salvia |
| D88 | 11 | NOS2 | Salvia |
| D89 | 10 | NOS2 | Salvia |
| D9 | 11 | NOS2 | Salvia |
| D90 | 12 | NOS2 | Salvia |
| D91 | 14 | NOS2 | Salvia |
| D92 | 11 | NOS2 | Salvia |
| D93 | 13 | NOS2 | Salvia |
| D94 | 10 | NOS2 | Salvia |
| D95 | 13 | NOS2 | Salvia |
| D96 | 13 | NOS2 | Salvia |
| D97 | 14 | NOS2 | Salvia |
| D98 | 10 | NOS2 | Salvia |
| DS010 | 13 | NOS2 | Salvia/Notoginseng |
| DS011 | 12 | NOS2 | Salvia/Notoginseng |
| DS3 | 9 | NOS2 | Salvia/Notoginseng |
| DS6 | 9 | NOS2 | Salvia/Notoginseng |
| DS7 | 13 | NOS2 | Salvia/Notoginseng |
| DS8 | 13 | NOS2 | Salvia/Notoginseng |
| DS9 | 11 | NOS2 | Salvia/Notoginseng |
| S1 | 14 | NOS2 | Notoginseng |
| S11 | 9 | NOS2 | Notoginseng |
| S114 | 14 | NOS2 | Notoginseng |
| S115 | 12 | NOS2 | Notoginseng |
| S116 | 12 | NOS2 | Notoginseng |
| S117 | 12 | NOS2 | Notoginseng |
| S118 | 11 | NOS2 | Notoginseng |
| S119 | 10 | NOS2 | Notoginseng |
| S12 | 15 | NOS2 | Notoginseng |
| S120 | 9 | NOS2 | Notoginseng |
| S121 | 13 | NOS2 | Notoginseng |
| S122 | 14 | NOS2 | Notoginseng |
| S123 | 13 | NOS2 | Notoginseng |
| S124 | 11 | NOS2 | Notoginseng |
| S125 | 13 | NOS2 | Notoginseng |
| S126 | 12 | NOS2 | Notoginseng |
| S127 | 11 | NOS2 | Notoginseng |
| S128 | 15 | NOS2 | Notoginseng |
| S132 | 9 | NOS2 | Notoginseng |
| S133 | 12 | NOS2 | Notoginseng |
| S134 | 13 | NOS2 | Notoginseng |
| S136 | 11 | NOS2 | Notoginseng |
| S137 | 11 | NOS2 | Notoginseng |
| S138 | 13 | NOS2 | Notoginseng |
| S139 | 13 | NOS2 | Notoginseng |
| S14 | 11 | NOS2 | Notoginseng |
| S140 | 13 | NOS2 | Notoginseng |
| S141 | 9 | NOS2 | Notoginseng |
| S142 | 9 | NOS2 | Notoginseng |
| S143 | 11 | NOS2 | Notoginseng |
| S147 | 10 | NOS2 | Notoginseng |
| S148 | 9 | NOS2 | Notoginseng |
| S15 | 10 | NOS2 | Notoginseng |
| S152 | 12 | NOS2 | Notoginseng |
| S153 | 13 | NOS2 | Notoginseng |
| S154 | 12 | NOS2 | Notoginseng |
| S155 | 10 | NOS2 | Notoginseng |
| S156 | 12 | NOS2 | Notoginseng |
| S16 | 12 | NOS2 | Notoginseng |
| S160 | 10 | NOS2 | Notoginseng |
| S163 | 12 | NOS2 | Notoginseng |
| S164 | 13 | NOS2 | Notoginseng |
| S165 | 12 | NOS2 | Notoginseng |
| S166 | 10 | NOS2 | Notoginseng |
| S168 | 9 | NOS2 | Notoginseng |
| S17 | 11 | NOS2 | Notoginseng |
| S170 | 16 | NOS2 | Notoginseng |
| S171 | 15 | NOS2 | Notoginseng |
| S172 | 15 | NOS2 | Notoginseng |
| S173 | 11 | NOS2 | Notoginseng |
| S174 | 12 | NOS2 | Notoginseng |
| S175 | 13 | NOS2 | Notoginseng |
| S176 | 13 | NOS2 | Notoginseng |
| S177 | 15 | NOS2 | Notoginseng |
| S179 | 10 | NOS2 | Notoginseng |
| S18 | 10 | NOS2 | Notoginseng |
| S181 | 13 | NOS2 | Notoginseng |
| S182 | 12 | NOS2 | Notoginseng |
| S183 | 13 | NOS2 | Notoginseng |
| S184 | 13 | NOS2 | Notoginseng |
| S185 | 13 | NOS2 | Notoginseng |
| S186 | 14 | NOS2 | Notoginseng |
| S187 | 13 | NOS2 | Notoginseng |
| S188 | 13 | NOS2 | Notoginseng |
| S189 | 13 | NOS2 | Notoginseng |
| S19 | 10 | NOS2 | Notoginseng |
| S190 | 13 | NOS2 | Notoginseng |
| S191 | 10 | NOS2 | Notoginseng |
| S192 | 10 | NOS2 | Notoginseng |
| S193 | 9 | NOS2 | Notoginseng |
| S195 | 13 | NOS2 | Notoginseng |
| S196 | 9 | NOS2 | Notoginseng |
| S197 | 10 | NOS2 | Notoginseng |
| S198 | 15 | NOS2 | Notoginseng |
| S199 | 10 | NOS2 | Notoginseng |
| S20 | 15 | NOS2 | Notoginseng |
| S201 | 13 | NOS2 | Notoginseng |
| S202 | 12 | NOS2 | Notoginseng |
| S203 | 13 | NOS2 | Notoginseng |
| S204 | 13 | NOS2 | Notoginseng |
| S205 | 13 | NOS2 | Notoginseng |
| S206 | 13 | NOS2 | Notoginseng |
| S207 | 13 | NOS2 | Notoginseng |
| S208 | 11 | NOS2 | Notoginseng |
| S209 | 11 | NOS2 | Notoginseng |
| S21 | 14 | NOS2 | Notoginseng |
| S210 | 10 | NOS2 | Notoginseng |
| S211 | 9 | NOS2 | Notoginseng |
| S212 | 11 | NOS2 | Notoginseng |
| S213 | 11 | NOS2 | Notoginseng |
| S214 | 11 | NOS2 | Notoginseng |
| S215 | 10 | NOS2 | Notoginseng |
| S216 | 14 | NOS2 | Notoginseng |
| S217 | 12 | NOS2 | Notoginseng |
| S218 | 11 | NOS2 | Notoginseng |
| S22 | 12 | NOS2 | Notoginseng |
| S220 | 12 | NOS2 | Notoginseng |
| S221 | 12 | NOS2 | Notoginseng |
| S222 | 11 | NOS2 | Notoginseng |
| S223 | 13 | NOS2 | Notoginseng |
| S224 | 11 | NOS2 | Notoginseng |
| S225 | 11 | NOS2 | Notoginseng |
| S226 | 13 | NOS2 | Notoginseng |
| S227 | 11 | NOS2 | Notoginseng |
| S228 | 14 | NOS2 | Notoginseng |
| S229 | 11 | NOS2 | Notoginseng |
| S23 | 13 | NOS2 | Notoginseng |
| S230 | 11 | NOS2 | Notoginseng |
| S231 | 14 | NOS2 | Notoginseng |
| S232 | 11 | NOS2 | Notoginseng |
| S233 | 15 | NOS2 | Notoginseng |
| S234 | 13 | NOS2 | Notoginseng |
| S235 | 11 | NOS2 | Notoginseng |
| S236 | 11 | NOS2 | Notoginseng |
| S237 | 13 | NOS2 | Notoginseng |
| S238 | 11 | NOS2 | Notoginseng |
| S239 | 13 | NOS2 | Notoginseng |
| S24 | 11 | NOS2 | Notoginseng |
| S240 | 11 | NOS2 | Notoginseng |
| S241 | 13 | NOS2 | Notoginseng |
| S242 | 11 | NOS2 | Notoginseng |
| S243 | 13 | NOS2 | Notoginseng |
| S244 | 12 | NOS2 | Notoginseng |
| S245 | 10 | NOS2 | Notoginseng |
| S246 | 12 | NOS2 | Notoginseng |
| S247 | 10 | NOS2 | Notoginseng |
| S248 | 10 | NOS2 | Notoginseng |
| S249 | 12 | NOS2 | Notoginseng |
| S25 | 13 | NOS2 | Notoginseng |
| S250 | 12 | NOS2 | Notoginseng |
| S251 | 12 | NOS2 | Notoginseng |
| S252 | 10 | NOS2 | Notoginseng |
| S253 | 12 | NOS2 | Notoginseng |
| S254 | 10 | NOS2 | Notoginseng |
| S255 | 14 | NOS2 | Notoginseng |
| S256 | 11 | NOS2 | Notoginseng |
| S258 | 9 | NOS2 | Notoginseng |
| S259 | 10 | NOS2 | Notoginseng |
| S26 | 13 | NOS2 | Notoginseng |
| S260 | 11 | NOS2 | Notoginseng |
| S261 | 11 | NOS2 | Notoginseng |
| S266 | 12 | NOS2 | Notoginseng |
| S271 | 11 | NOS2 | Notoginseng |
| S273 | 13 | NOS2 | Notoginseng |
| S274 | 11 | NOS2 | Notoginseng |
| S275 | 11 | NOS2 | Notoginseng |
| S277 | 14 | NOS2 | Notoginseng |
| S278 | 13 | NOS2 | Notoginseng |
| S28 | 12 | NOS2 | Notoginseng |
| S283 | 15 | NOS2 | Notoginseng |
| S287 | 15 | NOS2 | Notoginseng |
| S288 | 12 | NOS2 | Notoginseng |
| S289 | 10 | NOS2 | Notoginseng |
| S29 | 10 | NOS2 | Notoginseng |
| S290 | 9 | NOS2 | Notoginseng |
| S291 | 11 | NOS2 | Notoginseng |
| S292 | 10 | NOS2 | Notoginseng |
| S293 | 9 | NOS2 | Notoginseng |
| S294 | 10 | NOS2 | Notoginseng |
| S295 | 11 | NOS2 | Notoginseng |
| S298 | 10 | NOS2 | Notoginseng |
| S30 | 9 | NOS2 | Notoginseng |
| S300 | 10 | NOS2 | Notoginseng |
| S303 | 12 | NOS2 | Notoginseng |
| S307 | 9 | NOS2 | Notoginseng |
| S309 | 13 | NOS2 | Notoginseng |
| S31 | 15 | NOS2 | Notoginseng |
| S310 | 12 | NOS2 | Notoginseng |
| S314 | 9 | NOS2 | Notoginseng |
| S315 | 10 | NOS2 | Notoginseng |
| S318 | 11 | NOS2 | Notoginseng |
| S32 | 11 | NOS2 | Notoginseng |
| S325 | 9 | NOS2 | Notoginseng |
| S37 | 11 | NOS2 | Notoginseng |
| S38 | 9 | NOS2 | Notoginseng |
| S40 | 13 | NOS2 | Notoginseng |
| S41 | 13 | NOS2 | Notoginseng |
| S42 | 11 | NOS2 | Notoginseng |
| S45 | 9 | NOS2 | Notoginseng |
| S47 | 12 | NOS2 | Notoginseng |
| S48 | 10 | NOS2 | Notoginseng |
| S50 | 12 | NOS2 | Notoginseng |
| S52 | 13 | NOS2 | Notoginseng |
| S57 | 11 | NOS2 | Notoginseng |
| S58 | 11 | NOS2 | Notoginseng |
| S63 | 12 | NOS2 | Notoginseng |
| S65 | 12 | NOS2 | Notoginseng |
| S66 | 12 | NOS2 | Notoginseng |
| S68 | 9 | NOS2 | Notoginseng |
| S7 | 9 | NOS2 | Notoginseng |
| S76 | 10 | NOS2 | Notoginseng |
| S9 | 16 | NOS2 | Notoginseng |
| B11 | 9 | NOS3 | Borneol |
| B22 | 10 | NOS3 | Borneol |
| B25 | 9 | NOS3 | Borneol |
| B4 | 11 | NOS3 | Borneol |
| BD6 | 12 | NOS3 | Borneol/Salvia |
| BD7 | 9 | NOS3 | Borneol/Salvia |
| BD8 | 9 | NOS3 | Borneol/Salvia |
| BS1 | 10 | NOS3 | Borneol/Notoginseng |
| BS3 | 10 | NOS3 | Borneol/Notoginseng |
| BS6 | 12 | NOS3 | Borneol/Notoginseng |
| BS8 | 10 | NOS3 | Borneol/Notoginseng |
| D1 | 10 | NOS3 | Salvia |
| D107 | 11 | NOS3 | Salvia |
| D11 | 10 | NOS3 | Salvia |
| D120 | 9 | NOS3 | Salvia |
| D121 | 9 | NOS3 | Salvia |
| D131 | 9 | NOS3 | Salvia |
| D132 | 10 | NOS3 | Salvia |
| D136 | 9 | NOS3 | Salvia |
| D146 | 9 | NOS3 | Salvia |
| D166 | 9 | NOS3 | Salvia |
| D195 | 14 | NOS3 | Salvia |
| D200 | 10 | NOS3 | Salvia |
| D209 | 10 | NOS3 | Salvia |
| D213 | 9 | NOS3 | Salvia |
| D215 | 9 | NOS3 | Salvia |
| D216 | 10 | NOS3 | Salvia |
| D222 | 9 | NOS3 | Salvia |
| D38 | 12 | NOS3 | Salvia |
| D42 | 9 | NOS3 | Salvia |
| D44 | 9 | NOS3 | Salvia |
| D45 | 9 | NOS3 | Salvia |
| D46 | 9 | NOS3 | Salvia |
| D47 | 9 | NOS3 | Salvia |
| D53 | 9 | NOS3 | Salvia |
| D57 | 10 | NOS3 | Salvia |
| D60 | 9 | NOS3 | Salvia |
| D63 | 10 | NOS3 | Salvia |
| D64 | 10 | NOS3 | Salvia |
| D75 | 9 | NOS3 | Salvia |
| D76 | 9 | NOS3 | Salvia |
| D80 | 9 | NOS3 | Salvia |
| D81 | 10 | NOS3 | Salvia |
| D9 | 9 | NOS3 | Salvia |
| D95 | 9 | NOS3 | Salvia |
| D96 | 9 | NOS3 | Salvia |
| DS010 | 9 | NOS3 | Salvia/Notoginseng |
| DS011 | 9 | NOS3 | Salvia/Notoginseng |
| DS3 | 11 | NOS3 | Salvia/Notoginseng |
| DS7 | 9 | NOS3 | Salvia/Notoginseng |
| DS8 | 9 | NOS3 | Salvia/Notoginseng |
| S1 | 11 | NOS3 | Notoginseng |
| S11 | 10 | NOS3 | Notoginseng |
| S117 | 10 | NOS3 | Notoginseng |
| S118 | 10 | NOS3 | Notoginseng |
| S12 | 12 | NOS3 | Notoginseng |
| S122 | 9 | NOS3 | Notoginseng |
| S128 | 14 | NOS3 | Notoginseng |
| S142 | 10 | NOS3 | Notoginseng |
| S145 | 10 | NOS3 | Notoginseng |
| S147 | 10 | NOS3 | Notoginseng |
| S148 | 10 | NOS3 | Notoginseng |
| S15 | 9 | NOS3 | Notoginseng |
| S151 | 9 | NOS3 | Notoginseng |
| S152 | 9 | NOS3 | Notoginseng |
| S153 | 12 | NOS3 | Notoginseng |
| S154 | 9 | NOS3 | Notoginseng |
| S156 | 10 | NOS3 | Notoginseng |
| S158 | 9 | NOS3 | Notoginseng |
| S170 | 11 | NOS3 | Notoginseng |
| S171 | 13 | NOS3 | Notoginseng |
| S172 | 14 | NOS3 | Notoginseng |
| S177 | 11 | NOS3 | Notoginseng |
| S18 | 10 | NOS3 | Notoginseng |
| S186 | 10 | NOS3 | Notoginseng |
| S198 | 9 | NOS3 | Notoginseng |
| S20 | 11 | NOS3 | Notoginseng |
| S21 | 10 | NOS3 | Notoginseng |
| S223 | 9 | NOS3 | Notoginseng |
| S226 | 9 | NOS3 | Notoginseng |
| S234 | 9 | NOS3 | Notoginseng |
| S237 | 9 | NOS3 | Notoginseng |
| S239 | 9 | NOS3 | Notoginseng |
| S241 | 9 | NOS3 | Notoginseng |
| S243 | 9 | NOS3 | Notoginseng |
| S255 | 9 | NOS3 | Notoginseng |
| S260 | 9 | NOS3 | Notoginseng |
| S261 | 11 | NOS3 | Notoginseng |
| S274 | 10 | NOS3 | Notoginseng |
| S277 | 12 | NOS3 | Notoginseng |
| S280 | 9 | NOS3 | Notoginseng |
| S283 | 11 | NOS3 | Notoginseng |
| S287 | 9 | NOS3 | Notoginseng |
| S288 | 9 | NOS3 | Notoginseng |
| S289 | 9 | NOS3 | Notoginseng |
| S29 | 9 | NOS3 | Notoginseng |
| S290 | 10 | NOS3 | Notoginseng |
| S292 | 11 | NOS3 | Notoginseng |
| S293 | 9 | NOS3 | Notoginseng |
| S300 | 12 | NOS3 | Notoginseng |
| S303 | 12 | NOS3 | Notoginseng |
| S304 | 11 | NOS3 | Notoginseng |
| S307 | 12 | NOS3 | Notoginseng |
| S327 | 9 | NOS3 | Notoginseng |
| S4 | 11 | NOS3 | Notoginseng |
| S44 | 9 | NOS3 | Notoginseng |
| S52 | 9 | NOS3 | Notoginseng |
| S9 | 9 | NOS3 | Notoginseng |
| D41 | 9 | PRKCB | Salvia |
| S170 | 10 | PRKCB | Notoginseng |
| S9 | 9 | PRKCB | Notoginseng |
| D1 | 9 | SERPINE1 | Salvia |
| D120 | 9 | SERPINE1 | Salvia |
| D121 | 9 | SERPINE1 | Salvia |
| D172 | 9 | SERPINE1 | Salvia |
| D183 | 9 | SERPINE1 | Salvia |
| D209 | 10 | SERPINE1 | Salvia |
| D213 | 10 | SERPINE1 | Salvia |
| D216 | 16 | SERPINE1 | Salvia |
| S258 | 9 | SERPINE1 | Notoginseng |
| S307 | 10 | SERPINE1 | Notoginseng |
| S314 | 9 | SERPINE1 | Notoginseng |
| S320 | 9 | SERPINE1 | Notoginseng |
| B26 | 9 | SLC2A1 | Borneol |
| B38 | 10 | SLC2A1 | Borneol |
| B39 | 10 | SLC2A1 | Borneol |
| B8 | 10 | SLC2A1 | Borneol |
| BD1 | 10 | SLC2A1 | Borneol/Salvia |
| BD8 | 10 | SLC2A1 | Borneol/Salvia |
| BDS1 | 9 | SLC2A1 | Borneol/Salvia/Notoginseng |
| BS2 | 11 | SLC2A1 | Borneol/Notoginseng |
| BS3 | 10 | SLC2A1 | Borneol/Notoginseng |
| BS4 | 11 | SLC2A1 | Borneol/Notoginseng |
| BS6 | 16 | SLC2A1 | Borneol/Notoginseng |
| D10 | 9 | SLC2A1 | Salvia |
| D107 | 11 | SLC2A1 | Salvia |
| D11 | 12 | SLC2A1 | Salvia |
| D112 | 10 | SLC2A1 | Salvia |
| D12 | 11 | SLC2A1 | Salvia |
| D133 | 10 | SLC2A1 | Salvia |
| D134 | 14 | SLC2A1 | Salvia |
| D136 | 10 | SLC2A1 | Salvia |
| D137 | 12 | SLC2A1 | Salvia |
| D138 | 11 | SLC2A1 | Salvia |
| D140 | 10 | SLC2A1 | Salvia |
| D141 | 10 | SLC2A1 | Salvia |
| D142 | 9 | SLC2A1 | Salvia |
| D143 | 10 | SLC2A1 | Salvia |
| D146 | 10 | SLC2A1 | Salvia |
| D155 | 9 | SLC2A1 | Salvia |
| D156 | 11 | SLC2A1 | Salvia |
| D157 | 11 | SLC2A1 | Salvia |
| D158 | 9 | SLC2A1 | Salvia |
| D195 | 9 | SLC2A1 | Salvia |
| D20 | 9 | SLC2A1 | Salvia |
| D213 | 9 | SLC2A1 | Salvia |
| D216 | 9 | SLC2A1 | Salvia |
| D233 | 9 | SLC2A1 | Salvia |
| D3 | 11 | SLC2A1 | Salvia |
| D33 | 11 | SLC2A1 | Salvia |
| D35 | 9 | SLC2A1 | Salvia |
| D36 | 9 | SLC2A1 | Salvia |
| D37 | 9 | SLC2A1 | Salvia |
| D38 | 10 | SLC2A1 | Salvia |
| D39 | 9 | SLC2A1 | Salvia |
| D4 | 9 | SLC2A1 | Salvia |
| D56 | 10 | SLC2A1 | Salvia |
| D57 | 9 | SLC2A1 | Salvia |
| D65 | 11 | SLC2A1 | Salvia |
| D66 | 10 | SLC2A1 | Salvia |
| D76 | 9 | SLC2A1 | Salvia |
| D77 | 11 | SLC2A1 | Salvia |
| D78 | 12 | SLC2A1 | Salvia |
| D79 | 9 | SLC2A1 | Salvia |
| D80 | 9 | SLC2A1 | Salvia |
| D81 | 10 | SLC2A1 | Salvia |
| D88 | 9 | SLC2A1 | Salvia |
| D92 | 9 | SLC2A1 | Salvia |
| D93 | 9 | SLC2A1 | Salvia |
| DS011 | 14 | SLC2A1 | Salvia/Notoginseng |
| S115 | 10 | SLC2A1 | Notoginseng |
| S116 | 12 | SLC2A1 | Notoginseng |
| S117 | 11 | SLC2A1 | Notoginseng |
| S118 | 10 | SLC2A1 | Notoginseng |
| S12 | 9 | SLC2A1 | Notoginseng |
| S122 | 14 | SLC2A1 | Notoginseng |
| S128 | 10 | SLC2A1 | Notoginseng |
| S132 | 9 | SLC2A1 | Notoginseng |
| S14 | 10 | SLC2A1 | Notoginseng |
| S145 | 9 | SLC2A1 | Notoginseng |
| S146 | 12 | SLC2A1 | Notoginseng |
| S151 | 9 | SLC2A1 | Notoginseng |
| S155 | 10 | SLC2A1 | Notoginseng |
| S160 | 10 | SLC2A1 | Notoginseng |
| S165 | 11 | SLC2A1 | Notoginseng |
| S17 | 11 | SLC2A1 | Notoginseng |
| S170 | 11 | SLC2A1 | Notoginseng |
| S171 | 12 | SLC2A1 | Notoginseng |
| S172 | 10 | SLC2A1 | Notoginseng |
| S176 | 9 | SLC2A1 | Notoginseng |
| S177 | 9 | SLC2A1 | Notoginseng |
| S183 | 9 | SLC2A1 | Notoginseng |
| S184 | 9 | SLC2A1 | Notoginseng |
| S189 | 9 | SLC2A1 | Notoginseng |
| S19 | 9 | SLC2A1 | Notoginseng |
| S198 | 9 | SLC2A1 | Notoginseng |
| S20 | 12 | SLC2A1 | Notoginseng |
| S203 | 9 | SLC2A1 | Notoginseng |
| S204 | 9 | SLC2A1 | Notoginseng |
| S261 | 9 | SLC2A1 | Notoginseng |
| S263 | 9 | SLC2A1 | Notoginseng |
| S264 | 10 | SLC2A1 | Notoginseng |
| S274 | 14 | SLC2A1 | Notoginseng |
| S275 | 11 | SLC2A1 | Notoginseng |
| S277 | 13 | SLC2A1 | Notoginseng |
| S278 | 9 | SLC2A1 | Notoginseng |
| S28 | 10 | SLC2A1 | Notoginseng |
| S280 | 11 | SLC2A1 | Notoginseng |
| S281 | 10 | SLC2A1 | Notoginseng |
| S282 | 13 | SLC2A1 | Notoginseng |
| S283 | 14 | SLC2A1 | Notoginseng |
| S287 | 11 | SLC2A1 | Notoginseng |
| S290 | 9 | SLC2A1 | Notoginseng |
| S296 | 9 | SLC2A1 | Notoginseng |
| S298 | 14 | SLC2A1 | Notoginseng |
| S299 | 13 | SLC2A1 | Notoginseng |
| S30 | 10 | SLC2A1 | Notoginseng |
| S300 | 11 | SLC2A1 | Notoginseng |
| S303 | 10 | SLC2A1 | Notoginseng |
| S307 | 9 | SLC2A1 | Notoginseng |
| S315 | 9 | SLC2A1 | Notoginseng |
| S327 | 9 | SLC2A1 | Notoginseng |
| S47 | 11 | SLC2A1 | Notoginseng |
| S48 | 9 | SLC2A1 | Notoginseng |
| S65 | 10 | SLC2A1 | Notoginseng |
| S66 | 10 | SLC2A1 | Notoginseng |
| S9 | 11 | SLC2A1 | Notoginseng |
| DS011 | 13 | VCAM1 | Salvia/Notoginseng |
| S307 | 13 | VCAM1 | Notoginseng |
